# The *Ixodes scapularis* Symbiont *Rickettsia buchneri* Inhibits Growth of Pathogenic Rickettsiaceae in Tick Cells: Implications for Vector Competence

**DOI:** 10.3389/fvets.2021.748427

**Published:** 2022-01-06

**Authors:** Benjamin Cull, Nicole Y. Burkhardt, Xin-Ru Wang, Cody J. Thorpe, Jonathan D. Oliver, Timothy J. Kurtti, Ulrike G. Munderloh

**Affiliations:** ^1^Department of Entomology, College of Food, Agricultural, and Natural Resource Sciences, University of Minnesota, Saint Paul, MN, United States; ^2^Division of Environmental Health Sciences, School of Public Health, University of Minnesota, Minneapolis, MN, United States

**Keywords:** *Ixodes scapularis*, tick, *Rickettsia*, endosymbiont, antibiosis, interference, competition

## Abstract

*Ixodes scapularis* is the primary vector of tick-borne pathogens in North America but notably does not transmit pathogenic *Rickettsia* species. This tick harbors the transovarially transmitted endosymbiont *Rickettsia buchneri*, which is widespread in *I. scapularis* populations, suggesting that it confers a selective advantage for tick survival such as providing essential nutrients. The *R. buchneri* genome includes genes with similarity to those involved in antibiotic synthesis. There are two gene clusters not found in other Rickettsiaceae, raising the possibility that these may be involved in excluding pathogenic bacteria from the tick. This study explored whether the *R. buchneri* antibiotic genes might exert antibiotic effects on pathogens associated with *I. scapularis*. Markedly reduced infectivity and replication of the tick-borne pathogens *Anaplasma phagocytophilum, R. monacensis*, and *R. parkeri* were observed in IRE11 tick cells hosting *R. buchneri*. Using a fluorescent plate reader assay to follow infection dynamics revealed that the presence of *R. buchneri* in tick cells, even at low infection rates, inhibited the growth of *R. parkeri* by 86–100% relative to *R. buchneri*-free cells. In contrast, presence of the low-pathogenic species *R. amblyommatis* or the endosymbiont *R. peacockii* only partially reduced the infection and replication of *R. parkeri*. Addition of host-cell free *R. buchneri*, cell lysate of *R. buchneri-*infected IRE11, or supernatant from *R. buchneri-*infected IRE11 cultures had no effect on *R. parkeri* infection and replication in IRE11, nor did these treatments show any antibiotic effect against non-obligate intracellular bacteria *E. coli* and *S. aureus*. However, lysate from *R. buchneri-*infected IRE11 challenged with *R. parkeri* showed some inhibitory effect on *R. parkeri* infection of treated IRE11, suggesting that challenge by pathogenic rickettsiae may induce the antibiotic effect of *R. buchneri*. This research suggests a potential role of the endosymbiont in preventing other rickettsiae from colonizing *I. scapularis* and/or being transmitted transovarially. The confirmation that the observed inhibition is linked to *R. buchneri*'s antibiotic clusters requires further investigation but could have important implications for our understanding of rickettsial competition and vector competence of *I. scapularis* for rickettsiae.

## Introduction

The blacklegged tick *Ixodes scapularis* Say (Acari: Ixodidae) is the primary vector of zoonotic tick-borne pathogens in North America. It transmits seven pathogens, including those causing Lyme borreliosis (*Borrelia burgdorferi, Borrelia mayonii*), human anaplasmosis (*Anaplasma phagocytophilum*), and human babesiosis (*Babesia microti*) (1). Interestingly, in contrast to other major human-biting species of *Ixodes* in different parts of the world, *I. scapularis* does not transmit pathogenic *Rickettsia* species. *Ixodes ricinus* and *I. persulcatus*, the tick vectors primarily responsible for the transmission of *B. burgdorferi, A. phagocytophilum*, tick-borne encephalitis virus, and *Babesia* spp. in Europe and Asia, respectively, are commonly infected with *Rickettsia* spp. linked to human disease. Both *R. helvetica* and *R. monacensis* are commonly detected in *I. ricinus* across Europe (2–7), and various other rickettsiae including *R. raoultii* and *R. slovaca*, which are primarily vectored by other tick species, have also been identified in *I. ricinus* (3, 8). Meanwhile, *I. persulcatus* is infected with a wider range of *Rickettsia* spp., with *R. helvetica, R. raoultii, R. sibirica, R. heilongjiangensis*, and “*Candidatus* R. tarasevichiae” often detected in this tick species (3, 4, 9–15). In eastern Australia, *Ixodes holocyclus* is a vector of *R. australis*, the causative agent of Queensland tick typhus (16, 17).

**Figure 1:**
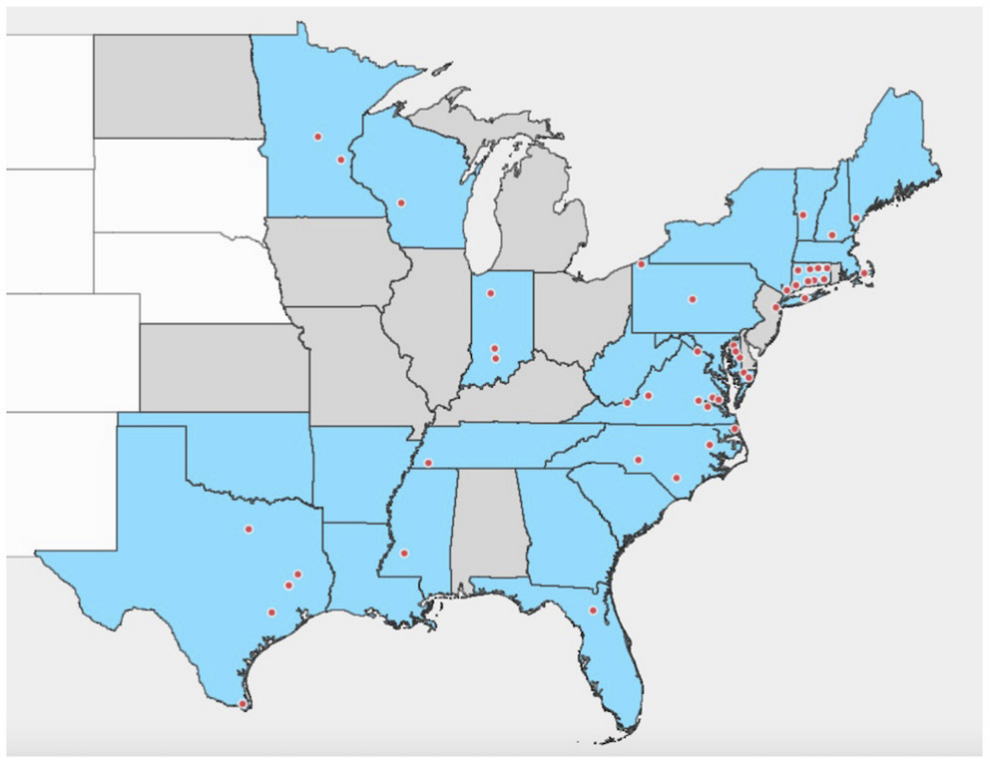
Distribution of detections of *R. buchneri* and “Rickettsial endosymbiont of *Ixodes scapularis*” in *Ixodes scapularis* in the United States of America. Shaded area indicates states with established populations of *Ixodes scapularis* in 2016 based on Eisen and Eisen (1). States shaded blue are those where *R. buchneri*/REIS has been detected in *I. scapularis*; records with county-level data are shown by dots. Based on data from references published 2007–2021 (18–23, 25–53).

Instead, *I. scapularis* hosts a rickettsial endosymbiont, *R. buchneri* (18), which dominates the tick microbiome, particularly in females where it typically constitutes almost 100% of the microbiome (19–24). These bacteria, formerly known as “rickettsial endosymbiont of *I. scapularis*” (REIS), have been detected in *I. scapularis* populations throughout its range (Figure 1) and are often present at high prevalence (18–23, 25–53), suggesting an established relationship between the tick and its endosymbiont. *Rickettsia buchneri* reside primarily in the ovaries of adult female ticks (18), although there is also some evidence of their presence in salivary glands (22, 52). The endosymbiont is transovarially transmitted and can be found in all life stages (22, 26), yet it is still unclear where it resides within adult males and immature stages, or what roles it may play in tick biology. The existence of genes in the endosymbiont encoding complete biosynthetic pathways for biotin and folate (54, 55) suggests that it may aid the tick by supplying essential nutrients lacking in blood. Phylogenetic analyses imply that *R. buchneri* is ancestral to the spotted fever group (SFG) rickettsiae (54), which contains the majority of tick-transmitted *Rickettsia* species; *R. buchneri* is most closely related to *R. monacensis* from *I. ricinus*, and the rickettsial endosymbiont of *Ixodes pacificus*, “*R. monacensis*” strain Humboldt (18, 56). Like other Rickettsiales, the SFG rickettsiae are obligate intracellular Gram-negative bacteria (57).

Cases of SFG rickettsioses are on the rise in the United States (58). While human cases of severe illness due to infection with *R. rickettsii* (Rocky Mountain spotted fever) appear to be rare, there have been increases in cases of milder spotted fever, thought to be primarily due to infection with other less pathogenic *Rickettsia* species and the geographic expansion of the lone star tick *Amblyomma americanum* (59). This tick commonly bites humans and is a potential vector of both *R. rickettsii* (60, 61) and *R. parkeri* (62), which causes a relatively mild eschar-associated rickettsiosis (63). Additionally, *R. amblyommatis*, originally considered an endosymbiont of *A. americanum* and highly prevalent in this tick, has been linked to mild disease (64–66), so it may also be contributing. The main vector of *R. parkeri*, the Gulf Coast tick *A. maculatum*, is also expanding its distribution (67), and populations are increasingly being found in more northerly US states (68, 69). The distribution of *I. scapularis* overlaps in large parts of the country with those of tick species responsible for the transmission of pathogenic SFG *Rickettsia* spp., particularly *A. americanum* and *Dermacentor variabilis*. These ticks may share similar habitats and hosts, therefore making it possible for *I. scapularis* to come into contact with pathogenic *Rickettsia* species. However, field-collected *I. scapularis* are very rarely infected with *Rickettsia* species other than *R. buchneri*. This might suggest that the presence of *R. buchneri* plays a role in excluding other *Rickettsia* spp. from its tick host. Evidence of competition (or “interference”) between different *Rickettsia* species exists in other ticks; for example, the presence of the endosymbiont *R. peacockii* in the Rocky Mountain wood tick *Dermacentor andersoni* has been associated with reduced transovarial transmission of pathogenic *R. rickettsii* in the tick (70). In addition, infection of *D. variabilis* with either *R. montanensis* or *R. rhipicephali* prevented the transovarial transmission of the competing rickettsia in reciprocal challenge experiments (71), and *A. americanum* infected with *R. amblyommatis* were less likely to acquire *R. parkeri* than uninfected ticks (72). Similarly, while *A. americanum* larvae infected with *R. amblyommatis* were able to acquire *R. rickettsii*, its prevalence was significantly lower compared to that in *R. amblyommatis-*free larvae (73). Furthermore, milder symptoms were observed in guinea pigs infected with *R. rickettsii* by dually infected nymphs than those infected by *R. amblyommatis*-free nymphs (73), suggesting that *R. rickettsii* load was reduced by the presence of the additional *Rickettsia* species. In field studies, a high prevalence of “*Candidatus* Rickettsia andeanae” in *A. maculatum* populations was hypothesized to be linked to the exclusion of *R. parkeri* from these ticks (74). Mechanisms for the competition between *Rickettsia* species, or whether these might differ for endosymbiotic and pathogenic species, have not been elucidated.

Two genome sequences of *R. buchneri* are currently available. The REIS (Wikel) genome was extracted from the genome sequence of *I. scapularis* from the Wikel colony (54), and the *R. buchneri* ISO7^T^ genome was sequenced from the *R. buchneri*-type strain isolated from the ovaries of a female *I. scapularis* removed from a dog in Minnesota (18). A gene cluster encoding aminoglycoside antibiotic biosynthesis machinery has been identified in *R. buchneri* (54), which is not present in other rickettsiae, and therefore antibiotic production might represent a mechanism by which *R. buchneri* is able to exclude pathogenic *Rickettsia* species from *I. scapularis*. Genes from the cluster were found to be highly similar to those of kanamycin and gentamicin synthesis gene clusters found in members of Actinobacteria and Firmicutes (54), yet to date no experimental studies have examined whether these genes are functional in *R. buchneri*. In this study, we report that an aminoglycoside biosynthesis gene cluster, almost identical to that described by Gillespie et al. (54), is also present in the *R. buchneri* ISO7^T^ genome, along with a second gene cluster encoding genes similar to those for polyketide and non-ribosomal peptide antibiotic synthesis, which appears to be only partially present in the REIS (Wikel) genome. Additionally, this study shows that genes from these clusters are actively transcribed and examines whether competition exists between *R. buchneri* and rickettsial pathogens associated with ticks, using *in vitro* experiments to provide preliminary evidence that the presence of the endosymbiont in tick cells has an inhibitory effect on the infection and replication of other intracellular bacteria.

## Materials and Methods

### Bioinformatic Analysis of *Rickettsia buchneri* Antibiotic Gene Clusters

Annotation of the sequenced *R. buchneri* ISO7^T^ genome [GenBank: JFKF01000000.1; (18)] identified the presence of two clusters of genes with similarity to bacterial genes for aminoglycoside, polyketide, and non-ribosomal peptide synthesis. This strain of *R. buchneri* was isolated from the ovaries of an *I. scapularis* female collected from a dog in Minnesota (18). To determine potential functions of proteins in the two putative antibiotic clusters, amino acid sequences from the *R. buchneri* ISO7^T^ genome were searched against other available sequences using the NCBI protein–protein BLAST algorithm, performed with default parameters. Putative protein function was determined by examining data for each protein in the InterPro database as well as performing literature searches. The gene clusters were also compared to the *R. buchneri* genome derived from the *I. scapularis* genome sequence, REIS (Wikel) [GenBank: CM000770.1; (54)]. Protein sequences obtained from annotated genomes were aligned using ClustalW (75) and MUSCLE (76) in MacVector version 18.1.5.

### Cell and Rickettsia Culture

Embryonic tick cell lines ISE6 (77), IRE11 (78), and AAE2 (79), derived from *I. scapularis, I. ricinus*, and *A. americanum*, respectively, were maintained at 34°C in L15C300 medium supplemented with 5% heat-inactivated fetal bovine serum (FBS), 5% tryptose phosphate broth (TPB), and 0.1% lipoprotein concentrate (LPC; MP Biomedicals, Irvine, CA, USA), adjusted to pH 7.2–7.5 with 1 M NaOH, as previously described (77). *Rickettsia buchneri* ISO7^T^ [(18); hereafter referred to as *Rb-*WT] and *R. buchneri* expressing GFPuv from the plasmid pRAM18dRGA [(80); hereafter referred to as *Rb-*GFPuv] were maintained in IRE11 at 28°C in a modified L15C300 medium containing 10% FBS, 5% TPB, 0.06% NaHCO_3_, 6 mM HEPES, and 0.1% LPC; pH was not adjusted. *Rickettsia parkeri* Tate's Hell expressing mKate from plasmid pRAM18dSFA (*Rp-*mKate) (80, 81), *R. peacockii-*GFPuv [pRAM18dSGK; (80)], *R. monacensis* IrR/Munich with mKate (pRAM18dSFA), and *A. phagocytophilum* HGE1 expressing mCherry from an intergenic Himar1 transposon insertion (82) (*A. phagocytophilum-*mCherry) were grown in ISE6 cells, and *R. amblyommatis* Darkwater (kindly supplied by Chris Paddock, CDC) were grown in AAE2 cells. Infected tick cell cultures were maintained at 34°C in L15C300 with 10% FBS, 5% TPB, 0.1% LPC, 0.25% NaHCO_3_, and 25 mM HEPES, adjusted to pH 7.5 (83). The infection level of cell cultures was assessed by Giemsa staining. Vero cells (African green monkey kidney) were grown in Gibco RPMI 1640 (Thermo Fisher, Waltham, MA, USA) supplemented with 10% FBS and 2 mM L-glutamine at 34°C following established methods (84). All cultures were grown in 25-cm^2^ culture flasks (CELLSTAR, Greiner Bio-One, Monroe, NC, USA).

Host cell-free bacteria were prepared as previously described (83); heavily infected tick cells were added to tubes containing rock tumbler grit (60/90 coarse silicon carbide, Lortone, Mukilteo, WA, USA), vortexed for 30 s, then passed through a 2 μm filter to remove cellular debris. Cell-free bacteria were then collected by centrifugation at 13,200 × g for 5 min at 4°C.

### Reverse Transcriptase PCR

Cell-free *Rb-*WT were prepared from infected IRE11 cultures as described above. Bacterial pellets were washed once in SPG buffer, then resuspended in 1 ml TRI Reagent (Sigma-Aldrich, St Louis, MO, USA), vortexed, and rested at room temperature for 10 min. Samples were centrifuged at 12,000 × g to remove particulates and the supernatant transferred to new tubes and mixed 1:2 with 100% ethanol, followed by vortexing. RNA was purified using a Direct-zol RNA Miniprep Kit (Zymo Research, Irvine, CA, USA). Contaminating DNA was removed by treating three times with Ambion TURBO DNA-free Kit (Thermo Fisher), followed by purification with an RNA Clean & Concentrator Kit (Zymo Research). Reverse transcriptase PCR (RT-PCR) was carried out using the Access RT-PCR system (Promega, Madison, WI, USA) in 25 μl reactions consisting of 5 × reaction buffer, 10 μM dNTPs, 10 μM of each primer, 0.5 μl Tf1 DNA polymerase, 14.5 μl nuclease-free water, and 1 μl sample. Nuclease-free water was used as a negative control, and no reverse transcriptase (no RT) controls included water instead of the DNA polymerase to confirm the absence of contaminating DNA. RT-PCR was performed in a Techne TC-312 Thermocycler with the following cycling conditions: 45 min at 45°C; 2 min at 94°C; 40 cycles of 30 s at 94°C, 1 min at 55°C or 58°C, and 2 min at 68°C; final extension 1 min at 68°C. Amplification of *HTH, lagD, ppsE_1, kanC*, and *btrB* was performed with an annealing temperature of 58°C, and amplification of *glycogen synthase, homoserine kinase*, and *l*g*rB* at 55°C. Primers used in this study are shown in Table 1. RT-PCR products were visualized on a 1.2% agarose gel stained with GelGreen (Biotium, Fremont, CA, USA).

**Table 1:** Primers used for the amplification of *Rickettsia buchneri* antibiotic cluster genes.

**Product name**	**Forward primer (5′-3′)**	**Reverse primer (5′-3′)**
HTH domain	AGC TGA TTT AGA AAG AAA GGC A	GAG GTA ACA TCA ATA CAG GGA AG
ppsE_1	CCT GGA GGT ATA AGA TCT GCT AAT G	GCT CCT TGT CCT GGG AAT AAA
lgrB	CTA CCG GAC AAC CTA AAG GAA TAG	CGG AAA CCT CGA ACC TTA ACT
btrB (choline dehydrogenase)	CGG GTT AAA TCC TTT CCC TAC TC	AGT AAC GAC AAG TCC CAT GTA AG
kanC	GGA GGA ATC CCA GGA AAC ATA G	CAA TGA GCA TAC CTA ACC CTA CA
lagD	AGT TCG GGT ATT GCC ACA TAT T	TGG TAT GCC ATA GGT AAG GAT TTC
Homoserine kinase	GTT CTA GCG CAA TAC CCT CTT	CGC GCA ATG TCC CAA ATA C
Glycogen synthase	TCC TGG CTA CTC GGT ACA TTA	CTC TGG CAA TAC GAC CAA CA
ppsE_1 (qRT-PCR)	ACG TAC TCC TAT GAA GCT CCG	GCT CCT TGT CCT GGG AAT AAA
lgrE (qRT-PCR)	TTT TCC CTT TCG CAG GTG GG	ACC CCA GAT ATT TTC CAC GTC C
btrB (qRT-PCR)	CGG GTT AAA TCC TTT CCC TAC TC	GCA TTC ATG CCT GCA AAA ATA G
kanC (qRT-PCR)	TAC ATG TCC AAG AGT ATG GCC G	AGC AGA GGC GAT AAA GCT AGT

### *In vitro* Competition Assays

Competition assays were set up in 24-well plates to compare the infection and replication of *R. monacensis-*mKate, *Rp-*mKate, and *A. phagocytophilum-*mCherry in tick cells with and without *Rb-*GFPuv infection. For the *R. monacensis* and *R. parkeri* experiments, three 24-well plates were prepared; the first contained uninfected IRE11, the second contained IRE11 heavily infected with *Rb*-GFPuv, and the third contained a mixture of infected and uninfected cells to give an approximate level of *Rb-*GFPuv infection of 45%. Cells (0.4 ml) were applied to each well, giving 1 × 10^6^ cells/well in the *R. monacensis* experiment or 3 × 10^5^ cells/well in the *R. parkeri* experiment. Plates were incubated in a humidified candle jar at 28°C. After 24 h, plates were infected with 250 μl fresh medium containing host cell-free *R. monacensis-*mKate or *Rp-*mKate, in 10-fold serial dilutions. Each dilution was applied to each plate in triplicate, and the remaining wells were used as negative controls. The number of mKate-positive colonies in the lowest-dilution wells was determined by fluorescent microscopy and used to extrapolate the number of rickettsiae in each dilution. Wells were observed on a Nikon Diaphot fluorescent microscope, and adhesion/invasion and replication were determined for each dilution on each plate by visualization of red fluorescent rickettsiae over 14 days. Additionally, the ability of *Rp*-mKate to infect IRE11 was assessed daily over the 14 days by observing at least 500 cells per well and determining the percentage of cells containing replicating fluorescent *R. parkeri*.

For *A. phagocytophilum* experiments, five 24-well plates were prepared; two contained uninfected IRE11, two contained IRE11 heavily infected with *Rb*-GFPuv, and the fifth contained a mixture of infected and uninfected cells to give an approximate level of *Rb-*GFPuv infection of 25%. Cells (0.5 ml) were applied to each well to give 1 × 10^6^ cells/well. One uninfected IRE11 plate and one infected IRE11 plate were incubated at 27°C in a humidified candle jar; the remaining three plates were incubated at 34°C with 4% CO_2_. After 24 h, plates were infected with 250 μl medium containing 10-fold serial dilutions of host cell-free *A. phagocytophilum*-mCherry, added to plates in triplicate. The number of *Anaplasma* in each dilution was estimated by testing each dilution in a sixth 24-well plate seeded with ISE6 cells and counting the number of mCherry-positive colonies in the lowest-dilution wells and then extrapolating to each dilution. Wells were observed on a Nikon Diaphot fluorescent microscope, and adhesion/invasion and replication were determined for each dilution on each plate by visualization of red fluorescent bacteria.

### Fluorescent Plate Reader Assays

**Figure 2:**
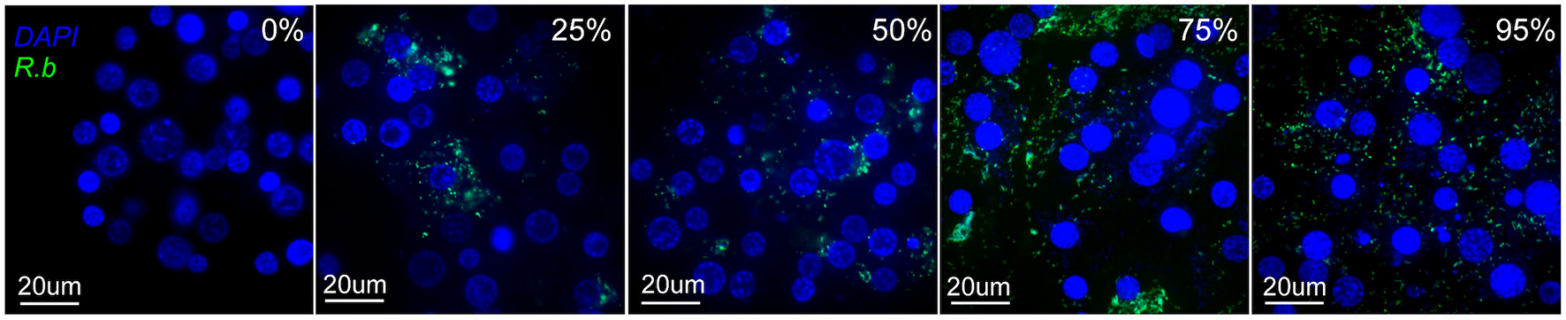
Images of IRE11 cells with different levels of *R. buchneri-*GFPuv infection. Live IRE11 cells were stained with NucBlue Live ReadyProbes Reagent (Hoechst 33342; Invitrogen) and spun onto slides with a Cytospin centrifuge, following Wang et al. (81). Images were captured on an Olympus BX61 DSU Confocal Microscope using a ×60 objective and a double-wavelength filter (DAPI; FITC). DNA shown in blue, GFPuv shown in green.

A fluorescent plate reader assay was used to measure the growth dynamics of fluorescent *Rickettsia* in IRE11 cells. Uninfected IRE11 and IRE11 heavily infected with *Rb-*GFPuv (>95% cells infected) were adjusted to 1 × 10^5^ cells/ml in fresh 10% FBS medium. Heavily infected and uninfected cells were then mixed to create additional populations with 25%, 50%, and 75% cells infected (Figure 2). A volume of 200 μl of each cell population was added in triplicate to wells of a clear-bottomed black-sided 96-well plate (Falcon, Corning, NY, USA), with dH_2_O added between wells to prevent drying, and incubated at 28°C in a humidified candle jar for 24 h to allow cells to settle to the bottom of wells. Host cell-free *Rp-*mKate were resuspended in 1 ml fresh medium and enumerated on a Petroff–Hausser chamber. The cell-free bacteria were diluted to create 1,000:1, 100:1, and 10:1 multiplicity of infection (MOI) ratios and then added to IRE11 cells in 96-well plates in a volume of 10 μl. The plate was returned to the humidified candle jar and incubated at 28°C. Fluorescence readings were taken 24 h later and then every 24 h up to 14 days postinfection. Readings were taken at room temperature (~22–25°C) on a BioTek Synergy H1 microplate reader at excitation/emission 395/509 for GFPuv and 588/633 for mKate and adjusted to uninfected IRE11 to account for background fluorescence.

Plate reader experiments for *R. amblyommatis* used a similar protocol to the above, except that only uninfected and 25% and >95% infected cell populations were used, and only 1,000:1 and 10:1 *Rp-*mKate challenges were performed. Additionally, only mKate fluorescence was measured for these experiments. The *R. peacockii* experiments were carried out using the same protocol as for *Rb-*GFPuv. Prior to IRE11 plate experiments, cell-free *R. amblyommatis* and *R. peacockii* (grown in AAE2 and ISE6 cells, respectively) were transferred to IRE11 and cultured in modified L15C300 medium at 28°C for at least 2 weeks to adjust them to growing in these conditions. Both *Rickettsia* species were found to grow well in IRE11 cells.

To assess the effect of cell-free *R. buchneri* and lysate of *R. buchneri* on the replication of *Rp-*mKate, a 96-well plate was set up with 200 μl of uninfected IRE11 adjusted to 1 × 10^5^ cells/ml in fresh medium, as above. Cells were treated with either 50 μl medium (negative control), 50 μl of cell-free *Rb-*WT prepared from 2.5 × 10^5^ heavily infected IRE11, 50 μl of a 1:10 dilution of the cell-free *Rb-*WT, 50 μl cell lysate from 2.5 × 10^5^ IRE11 heavily infected with *Rb-*WT, or 50 μl cell lysate from 2.5 × 10^5^ uninfected IRE11. Lysates were prepared by sonicating cells on ice at full power for a total of 1 min (separated into 3 × 20-s bursts, with 20-s intervals resting on ice). After 2 h, cells were challenged with cell-free *Rp-*mKate at 1,000:1, 100:1, or 10:1, and the plate was incubated at 28°C in a humidified candle jar. mKate fluorescence was measured every 24 h for 14 days, as described above, and adjusted to uninfected, untreated IRE11.

To determine if *R. parkeri* could induce *R. buchneri* antibiosis activity, an additional plate reader assay was used to examine whether lysates from *R. buchneri* challenged with *Rp-*mKate for varying lengths of time exhibited inhibitory effects on the growth of *Rp-*mKate in IRE11 cells. Wells of a 6-well plate were seeded with 2 ml IRE11 75% infected with *Rb*-WT, at 1 × 10^5^/ml. One well served as a no challenge control, while to the remaining wells cell-free *Rp-*mKate at a ratio of 100:1 was added. The plate was incubated at 28°C in a candle jar. After 24 h, cells from the control and one of the challenged wells were collected, and rickettsiae were isolated from IRE11 as above. Cell pellets were then frozen at −70°C. At 48, 72, 120, and 168 h after infection, this was repeated for cells from each of the remaining wells. Lysates were prepared from the pellets by four freeze thaw cycles of −70 to 37°C. The rickettsial lysates from each treatment were resuspended in 120 μl medium, and then 10 μl was added to 12 wells of a 96-well plate, each containing 200 μl IRE11 at 1 × 10^5^/ml, prepared 24 h previously. Wells were then challenged with *Rp-*mKate at ratios of 1,000:1, 100:1, and 10:1. Medium without *Rp-*mKate was added to control wells for each treatment. The plate was incubated at 28°C in a humidified candle jar, and mKate fluorescence was measured every 24 h for 14 days, as described above.

### Antibiotic Susceptibility Assays

To test the antibiotic activity of *R. buchneri* against extracellular bacteria, antibiotic susceptibility testing was performed against *Escherichia coli* D21 and *Staphylococcus aureus* MN8 using disk diffusion assays. IRE11 infected with *Rb-*WT and uninfected IRE11 was pelleted by centrifugation at 350 × *g* for 6 min at 4°C, and the pellets were frozen at −70°C. Cell-free *Rb-*WT was prepared by vortexing with rock tumbler grit and filtration through a 2-μm filter, as described above, then the bacteria were pelleted by centrifugation at 13,600 × *g* for 7 min at 4°C, and the cell pellet frozen at −70°C. Lysates of the three samples were prepared by freeze-thawing (four cycles of −70 to 37°C) and then centrifugation at 13,600 × *g* for 5 min at 4°C. Bacterial or cell lysates were then resuspended in 50 μl medium, of which 30 μl was added to 50 μl of absolute methanol. A volume of 20 μl of each sample was then added to separate filter paper disks. Spectinomycin at 10 and 100 μg was added to two additional filter paper disks as positive controls. Disks were air-dried in a biosafety cabinet for 20–30 min and then placed onto Mueller–Hinton agar plates streaked with *E. coli* D21 or *S. aureus* MN8. Plates were incubated for 18 h at 37°C.

The disk diffusion assays were repeated using pellets of live IRE11, *Rb-*WT-infected IRE11 (25%, 50%, and >95% infected), and cell-free *Rb-*WT, as well as supernatant from IRE11 cultures at various levels of infection with *Rb-*WT (25, 50, >95%) or uninfected. Cells were pelleted by centrifugation at 350 × *g* for 6 min at 12°C, supernatant was removed to separate tubes, and then pellets were resuspended in 100 μl medium. A volume of 20 μl of resuspended cell pellets or 20 μl supernatant was added to filter paper disks. Spectinomycin was added to additional disks at 100 μg for positive controls. Disks were air-dried for 20-30 min in a biosafety cabinet, then applied to Mueller–Hinton agar plates streaked with *E. coli* D21 or *S. aureus* MN8. Plates were incubated for 18 h at 37°C.

To further test the antibiotic activity of supernatant from *Rb-*WT-infected IRE11 cultures against *R. parkeri* grown in mammalian cells, Vero cell cultures were grown at 34°C in 2.5 ml RPMI medium supplemented with 2.5 ml supernatant from either uninfected IRE11 or IRE11 heavily infected with *Rb-*WT. Cell-free *Rp-*mKate was added to Vero cultures and flasks checked daily for evidence of infection, which was assessed by the appearance of plaques in the cell layer as well as the timing and size of plaques.

### Expression of Antibiotic Genes in Response to *R. parkeri* Challenge

To determine whether the expression of antibiotic genes by *Rb-*WT was induced by challenge with *R. parkeri*, and to determine the time of maximal expression, a time course experiment was set up comparing unchallenged and *Rp-*mKate-challenged *Rb-*WT-infected IRE11. A 6-well plate was prepared with each well containing IRE11 75% infected with *Rb-*WT at 1 × 10^5^/ml in 2 ml modified L15C300 medium. One well served as a no challenge control, while to the remaining wells cell-free *Rp-*mKate at a ratio of 100:1 in 10 μl was added. The plate was incubated at 28°C in a candle jar. After 24 h, cells from the control well were collected and centrifuged for 2 min at 500 × *g*, and the cell pellet was resuspended in 750 μl RNAlater solution (Qiagen, Hilden, Germany) and stored at−20°C. Cells from the challenge wells were collected at 24, 48, 72, 120, and 168 h after infection, resuspended in RNAlater solution, and stored at −20°C. RNA isolation was performed using TRIzol–chloroform extraction. cDNA was then prepared using Takara RT PrimeScript Kit (Takara Bio, Kusatsu, Japan), after a 30-min treatment with a gDNA eraser at room temperature to remove contaminating DNA. Quantitative PCR (qPCR) was performed on an Agilent Mx3005P RT-PCR system using the Agilent Brilliant II SYBR Green Master Mix, using 10-μM primers against various targets from both gene clusters (Table 1) and 1 μl cDNA preparation. Since both *R. buchneri* and *R. parkeri* were present in samples, *I. scapularis GAPDH* was used as a reference gene. Nuclease-free water and no reverse transcriptase controls were included on each plate. Each sample was run in triplicate. Cycling conditions were 10 min at 95°C, followed by 40 cycles of 30 s at 95°C, 1 min at 55°C (*glycogen synthase, GAPDH*), or 52°C (*btrB, kanC, lgrE, ppsE_1*), 1 min at 72°C. Results were compared to *GAPDH* expression to adjust for total cDNA per sample. To measure primer efficiency, standard curves consisting of cDNA from *R. buchneri*-infected IRE11 were also added to reaction plates. To account for differences in primer efficiency, relative quantification was calculated with a PCR efficiency correction (85).

### Statistical Analysis

All statistical analyses were carried out using GraphPad Prism version 9.1.2. For plate reader experiments, the growth of *Rp-*mKate in IRE11 cultures infected with other *Rickettsia* or treated with lysates was analyzed using a two-way ANOVA with Dunnett's multiple-comparison test, using *Rp-*mKate growth in uninfected IRE11 wells as the control. Relative gene expression was analyzed with a two-way ANOVA with Dunnett's multiple-comparison test, comparing the expression in *Rp-*challenged groups to that in the unchallenged control group. Statistical significance was assigned when *p* < 0.05.

## Results

### Description and Expression of Antibiotic Gene Clusters in *Rickettsia buchneri*

**Figure 3:**
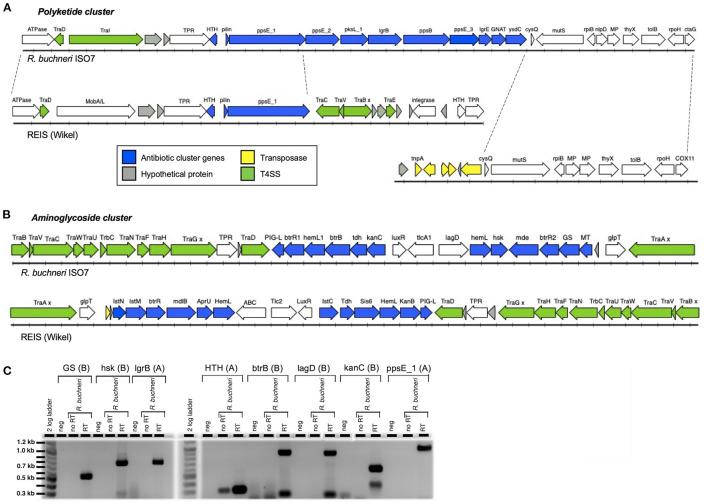
Antibiotic gene clusters in *Rickettsia buchneri*. Diagrams showing the gene arrangement of *R. buchneri* ISO7 antibiotic gene clusters containing polyketide **(A)** and aminoglycoside **(B)** synthesis genes, and neighboring regions of the genome, and their comparison with those in the REIS (Wikel) genome. GS—glycogen synthase; hsk—homoserine kinase; MT—methyltransferase; MP—metallopeptidase; x denotes a mutation resulting in a truncation. **(C)** RT-PCR of *R. buchneri* ISO7 RNA showing the transcription of selected genes from antibiotic clusters. The gene product and antibiotic cluster (A = polyketide, B = aminoglycoside) are shown above the wells of the gel. See Tables 2, 3 for additional information.

Analysis of the sequenced *R. buchneri* ISO7^T^ genome revealed that it contains two gene clusters encoding proteins similar to those involved in antibiotic synthesis. Neither gene cluster is present in other members of *Rickettsiaceae*. The first cluster contains eleven genes including those for polyketide and other non-ribosomal peptide synthesis enzymes (Table 2; Figure 3A). Biosynthesis pathways for these compounds (which include the penicillins, cyclosporin A, vancomycin, and erythromycin) involve large multi-modular enzymes that act as assembly lines for the catalysis of chain elongation and addition of modifications (86). These are usually clustered with additional genes encoding tailoring enzymes that further modify the resulting compound, for example by methylation or cyclization, and/or by releasing it from the assembly line (87, 88). Proteins in the *R. buchneri* polyketide cluster show similarity to sequences from the Gammaproteobacteria *Legionella israelensis, Erwinia amylovora, Pantoea ananatis*, and *Pectobacterium* spp. as well as Cyanobacteria (Supplementary Figure S1; Supplementary Data S1). Interestingly, one of the hypothetical proteins in the cluster appears to be a type IV pilin, whose sequence obtained no blastp hits except those from the two *R. buchneri* genomes. Only the first three genes in the cluster are conserved in the REIS (Wikel) genome, while the remaining genes are not present (Figure 3A; Table 2; Supplementary Figure S1). Instead, the region of the REIS genome downstream of *ppsE_1* encodes numerous *tra* genes (REIS_1815 = *traC*, REIS_1814 = *traV*, REIS_1813 = truncated *traB*, REIS_1810 = *traE*). The region upstream of the cluster is homologous in both genomes with a similar arrangement of genes (Figure 3A). Interestingly, the genes following *ysdC* in the *Rb* ISO7 genome are homologous to those in a different region of the REIS genome, with the next gene after *ysdC*, REISMN_1205, identical to REIS_1393 (Figure 3A). Notably the area upstream of REIS_1393 is heavily populated with transposase sequences, suggesting that a transposition event may have led to the loss of the remaining polyketide cluster genes in the REIS genome.

**Table 2:** Genes in *Rickettsia buchneri* putative polyketide synthesis cluster.

**Label**	**Locus tag**	**Accession**	**Length (aa)**	**Annotation^a^**
HTH domain^*^	REISMN_01150 (REIS_1819)	KDO03565.1	142	**Helix-turn-helix transcriptional regulator**
Hypothetical	REISMN_01155 (REIS_1817)	KDO03566.1	62	**hypothetical protein;** type IV pilin
ppsE_1^*^	REISMN_01160 (REIS_1816)	KDO03567.1	1448	**Beta-ketoacyl-acyl-carrier-protein synthase I**; type I polyketide synthase; erythronolide synthase; acyltransferase domain-containing protein
ppsE_2	REISMN_01165	KDO03568.1	636	**Beta-ketoacyl-acyl-carrier-protein synthase I**; type I polyketide synthase; SDR family NAD(P)-dependent oxidoreductase
pksL_1	REISMN_01170	KDO03569.1	522	**Polyketide synthase PksL;** SDR family NAD(P)-dependent oxidoreductase; type I polyketide synthase
lgrB^*^	REISMN_01175	KDO03570.1	630	**Linear gramicidin synthase subunit B;** non-ribosomal peptide synthetase
ppsB	REISMN_01180	KDO03571.1	878	**Plipastatin synthase subunit B;** non-ribosomal peptide synthetase
ppsE_3	REISMN_01185	KDO03572.1	554	**Beta-ketoacyl-acyl-carrier-protein synthase I;** polyketide synthase
lgrE	REISMN_01190	KDO03573.1	239	**Linear gramicidin dehydrogenase LgrE;** thioesterase
Hypothetical	REISMN_01195	KDO03574.1	265	**hypothetical protein;** GNAT family N-acetyltransferase
ysdC	REISMN_01200	KDO03575.1	392	**Putative aminopeptidase ysdC;** M42 family metallopeptidase

*Asterisks indicate genes with transcription confirmed by RT-PCR*.

*Locus tags for corresponding genes in the REIS (Wikel) genome are shown in brackets*.

a *Bold type indicates annotation recorded in the genome; regular type indicates additional annotations gained through protein BLAST searches*.

Gillespie et al. (54) also identified an additional polyketide synthase with a putative frameshift mutation (REIS_0330). This prompted an examination of the *Rb* ISO7 genome for additional polyketide synthase genes. A cluster of three were identified, and these are annotated as *pksL_2* (REISMN_07055), *pksR* (REISMN_07060), and *pksN* (REISMN_07065). Corresponding genes encoding identical proteins are annotated in the REIS (Wikel) RefSeq on GenBank, with corresponding locus tags REIS_RS15050 (hypothetical protein), REIS_RS10770 (methyltransferase), and REIS_RS01405 (KR domain-containing protein), respectively.

The second cluster contains fifteen genes, with eleven showing similarity to genes involved in aminoglycoside antibiotic synthesis, as well as genes coding for putative antibiotic exporters and an antibiotic resistance factor (Table 3; Figure 3B). This second antibiotic cluster was also identified in the REIS (Wikel) genome (54) and found to be associated with the *Rickettsiales*-amplified genetic element, RAGE-A. The aminoglycoside antibiotics include streptomycin, kanamycin, and gentamicin and are produced through complex biosynthetic pathways involving many enzymatic reactions; few of these pathways have been fully characterized (89–91). Proteins of the *R. buchneri* aminoglycoside cluster show similarity to those from antibiotic synthesis gene clusters from Actinobacteria and Firmicutes (54), while the putative multidrug exporter mde/mdlB, transcriptional regulator LuxR, and ABC transporter lagD show greater similarity to proteins from the Gammaproteobacteria (Supplementary Figure S2; Supplementary Data S2). Meanwhile, the nucleotide translocase tlcA1/tlc2 is related to those from other *Rickettsia* species (54). This cluster is conserved in both *R. buchneri* genomes (Figure 3B; Table 3), with the majority of sequences identical (Supplementary Figure S2).

**Table 3:** Genes in *Rickettsia buchneri* putative aminoglycoside synthesis cluster.

**Label**	**Locus tag**	**Accession**	**Length (aa)**	**Annotation^a^**
PIG-L (PIG-L)	REISMN_01820 (REIS_1505)	KDO03398.1	234	**GlcNAc-PI de-N-acetylase;** PIG-L family deacetylase
btrR_1 (KanB)	REISMN_01825 (REIS_1504)	KDO03399.1	421	**L-Glutamine:2-deoxy-scyllo-inosose aminotransferase;** DegT/DnrJ/EryC1/StrS family aminotransferase
hemL1 (HemL)	REISMN_01830 (REIS_1503)	KDO03400.1	420	**Glutamate-1-semialdehyde 2,1-aminomutase 1;** aminotransferase class III-fold pyridoxal phosphate dependent enzyme
btrB^*^ (Sis6)	REISMN_01835 (REIS_1502)	KDO03401.1	518	**Choline dehydrogenase;** GMC family oxidoreductase
tdh (Tdh)	REISMN_01840 (REIS_1501)	KDO03402.1	342	**L-Threonine 3-dehydrogenase**
kanC^*^ (IstC)	REISMN_01845 (REIS_1500)	KDO03403.1	383	**2-Deoxy-scyllo-inosose synthase**
luxR (LuxR)	REISMN_01850 (REIS_1499)	KDO03404.1	275	**luxR family transcriptional regulator**
tlcA1 (Tlc2)	REISMN_01855 (REIS_1498)	KDO03405.1	521	**ADP/ATP translocase 1**
lagD^*^ (ABC)	REISMN_01860 (REIS_1497)	KDO03406.1	605	**Lactococcin-G-processing and transport ATP-binding protein LagD;** ABC transporter ATP-binding protein/permease
hemL (HemL)	REISMN_01865 (REIS_1496)	KDO03407.1	435	**Glutamate-1-semialdehyde 2,1-aminomutase**
Homoserine kinase^*^ (AprU)	REISMN_01870 (REIS_1495)	KDO03408.1	342	**Homoserine kinase**
mde protein (mdlB)	REISMN_01875 (REIS_1494)	KDO03409.1	594	**Putative multidrug export ATP-binding/permease protein**
btrR_2 (btrR)	REISMN_01880 (REIS_1493)	KDO03410.1	392	**L-Glutamine:2-deoxy-scyllo-inosose aminotransferase;** DegT/DnrJ/EryC1/StrS family aminotransferase
Glycogen synthase^*^ (IstM)	REISMN_01885 (REIS_1492)	KDO03411.1	422	**Glycogen synthase**
Methyl transferase (IstN)	REISMN_01890 (REIS_1491)	KDO03412	262	**Hypothetical protein;** class I SAM-dependent methyltransferase

*Asterisks indicate genes with transcription confirmed by RT-PCR. Protein labels and locus tags for corresponding genes in the REIS (Wikel) genome are shown in brackets*.

a *Bold type indicates annotation recorded in the genome; regular type indicates additional annotations gained through protein BLAST searches*.

To determine whether these genes are actively transcribed by *R. buchneri*, eight genes from the two antibiotic clusters were selected for RT-PCR analysis (Tables 2, 3). Transcripts for all eight genes were detected, as indicated by bands of the expected sizes observed on gel electrophoresis (Figure 3C). Negative controls and no RT controls showed no products, except for *HTH* where a less robust band was seen, suggesting some DNA contamination, although there is clearly less amplification than in the RT well. Smaller bands observed in the *btrB, lagD*, and *kanC* reactions likely represent nonspecific products.

### *In vitro* Antibiosis Experiments

**Figure 4:**
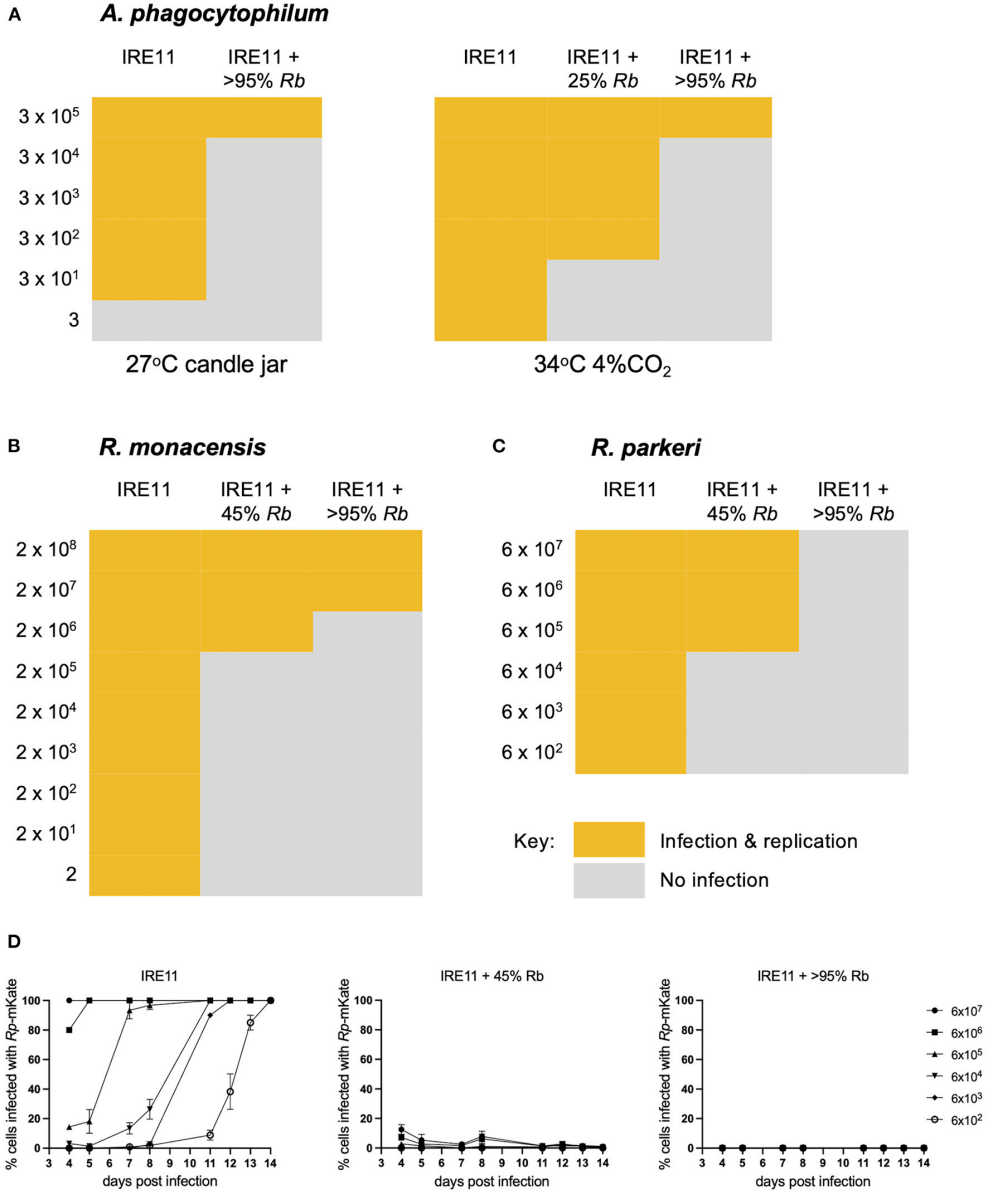
*Rickettsia buchneri* inhibits infection and replication of other tick-borne bacteria in tick cell culture. IRE11 cells infected with different levels of *R. buchneri-*GFPuv were infected with serial dilutions of red fluorescent *A. phagocytophilum* **(A)**, *R. monacensis* **(B)**, or *R. parkeri* **(C)**, and plates were monitored over 14 days for infection and replication, compared to uninfected IRE11 cells. **(D)** The percentage of IRE11 cells infected with *R. parkeri-*mKate was assessed by fluorescent microscopy. Measurements not taken on days 6, 9, and 10.

To determine whether *R. buchneri* might exhibit antibiosis against other bacteria infecting tick cells, cell-free red fluorescent *A. phagocytophilum, R. monacensis*, or *R. parkeri* was used to challenge tick cell cultures containing different levels of green fluorescent *R. buchneri*. Infectivity was measured 14 days after inoculating serial dilutions of cell-free rickettsiae/*Anaplasma* into replicated wells of a 24-well tissue culture plate seeded with *R. buchneri*-infected IRE11 cells. All three pathogens showed markedly reduced ability to infect and replicate in tick cell cultures infected with *R. buchneri* (Figure 4), and even a low level of infection with *Rb*-GFPuv led to a reduction in pathogen infection and replication. Fluorescent microscopy revealed that *R. monacensis* and *A. phagocytophilum* did not replicate in tick cells infected with *R. buchneri* (Supplementary Figure S3). Infectivity of *R. monacensis, R. parkeri*, or *A. phagocytophilum* in tick cell cultures infected with *R. buchneri* was reduced by 3–5 orders of magnitude compared to that in cells without *R. buchneri* (Figure 4), confirming that the presence of *R. buchneri* was inhibitory to the growth of other intracellular tick-borne bacteria. Furthermore, examining the percentage of infected cells by fluorescent microscopy during *Rp-*mKate infection showed that in IRE11 without *R. buchneri*, cells became completely infected at all dilutions over the 14-day period (Figure 4D). In contrast, *Rp-*mKate infection only reached low levels (<10%) in IRE11 cultures containing the endosymbiont in 45% cells, and almost no cells became infected with *Rp-*mKate in cultures with >95% cells harboring *R. buchneri*.

**Figure 5:**
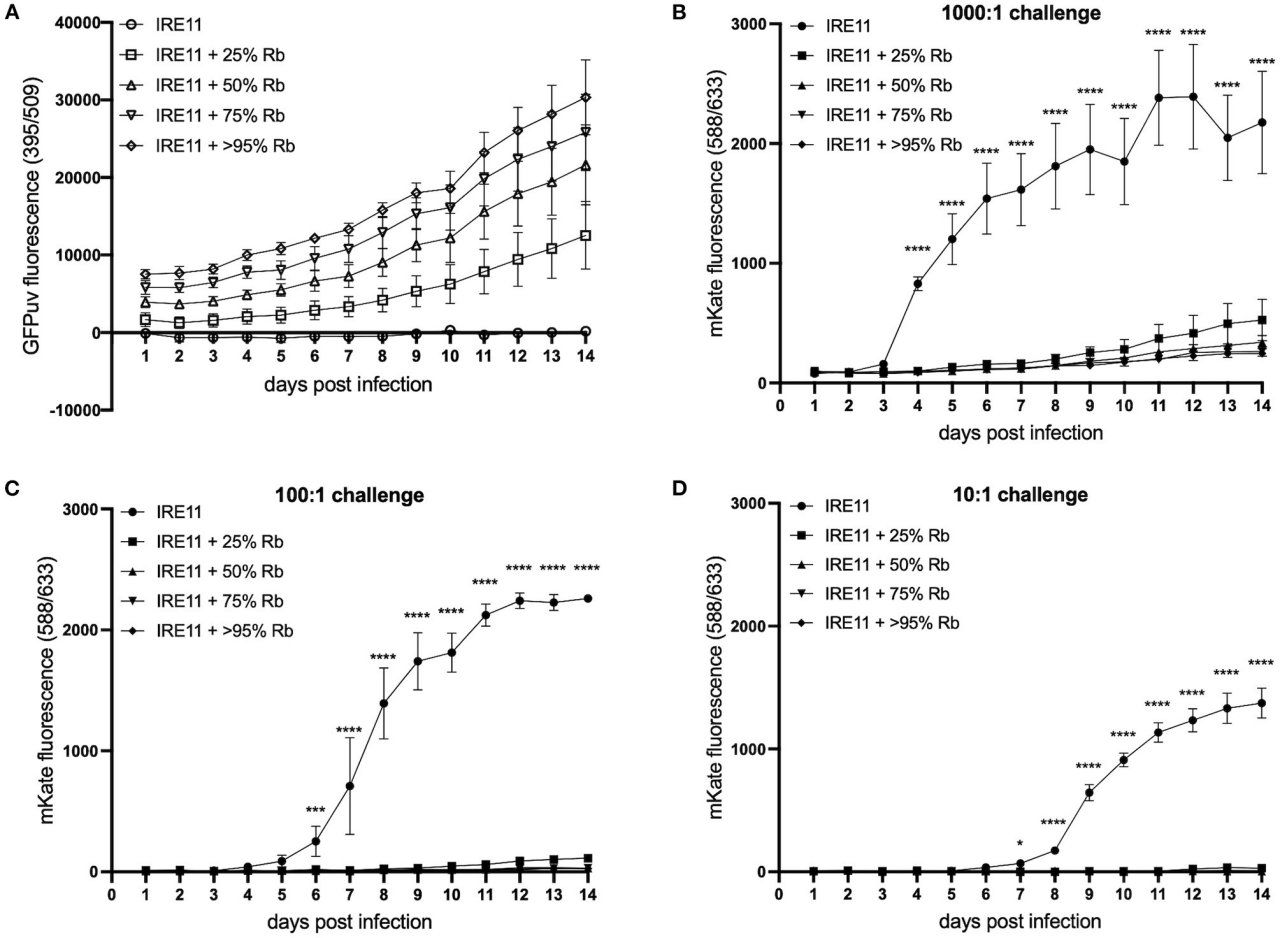
*Rickettsia buchneri* prevents infection and replication of *Rickettsia parkeri* in tick cell culture. Uninfected IRE11 and IRE11 infected with different levels of *R. buchneri-*GFPuv were challenged with different doses of *R. parkeri-*mKate. Rickettsial replication in IRE11 cells was monitored for 14 days by measuring GFPuv and mKate fluorescence on a microplate reader. **(A)** GFPuv fluorescence indicating replication of *R. buchneri-*GFPuv. **(B–D)** mKate fluorescence indicating growth of *R. parkeri-*mKate at challenge doses of 1,000:1 **(B)**, 100:1 **(C)**, and 10:1 **(D)**; lines show mean and error bars standard deviation of three replicate wells. Means were compared to the uninfected control IRE11 using a two-way ANOVA with Dunnett's multiple-comparison test; statistically significant values are marked by asterisks ^*^p < 0.05, ^***^p < 0.001, ^****^p < 0.0001. Data are representative of two independently performed experiments (see Supplementary Figure S4).

To further investigate the growth dynamics during coinfection, a fluorescent plate reader was used to measure the replication of *Rp-*mKate in IRE11 cells infected with *Rb*-GFPuv at levels of 25, 50, 75, and >95%, in comparison to uninfected IRE11. GFPuv measurement could clearly differentiate the various levels of infection with *Rb*-GFPuv and indicated a steady replication of the endosymbiont over the 14-day experiment (Figure 5A). No increases in GFPuv fluorescence were seen in uninfected IRE11. With a high (1,000:1) challenge, fluorescence from *Rp-*mKate growth indicated a rapid infection and replication of *Rp*-mKate in uninfected IRE11 from day 3 which then began to level off from day 6 onward (Figure 5B). In contrast, there were significantly lower rates of mKate fluorescence increase in IRE11 harboring *Rb-*GFPuv at all levels of infection from day 4 onward (*p* < 0.0001); compared to uninfected IRE11 at day 14, there was an 89% reduction in *Rp-*mKate in IRE11 with >95% *Rb*-GFPuv, 88% reduction in IRE11 with 75% *Rb*-GFPuv, 84% reduction in IRE11 with 50% *Rb-*GFPuv, and 76% reduction in IRE11 with 25% *Rb-*GFPuv. Similarly, 100:1 challenge of uninfected IRE11 with *Rp-*mKate resulted in a rapid increase in fluorescence from days 5 to 6, reaching a peak by day 12, indicating replication and spread of *Rp-*mKate in the cells (Figure 5C). However, in IRE11 with *Rb-*GFPuv, significant differences in mKate fluorescence were observed from day 6 (*p* < 0.001), and at day 14 the reduction was 99% in 50%, 75% and >95% infected cells, and 95% in IRE11 with 25% *Rb-*GFPuv. With a low challenge (10:1), mKate fluorescence increased from days 7 to 8 and reached its height at days 13–14 in uninfected IRE11, whereas in *Rb-*GFPuv-infected IRE11 mKate fluorescence was reduced by 99%-100% in 50%, 75%, and >95% infected cells, and by 98% in 25% infected cells (Figure 5D), with significant differences seen from day 8 (*p* < 0.0001) compared to IRE11 without *Rb-*GFPuv. No changes in mKate fluorescence were observed in control wells to which *Rp-*mKate was not added (data not shown). Together, these results suggest that the presence of *R. buchneri* in IRE11 has a significant inhibitory effect on the ability of *R. parkeri* to successfully infect and replicate in the culture.

**Figure 6:**
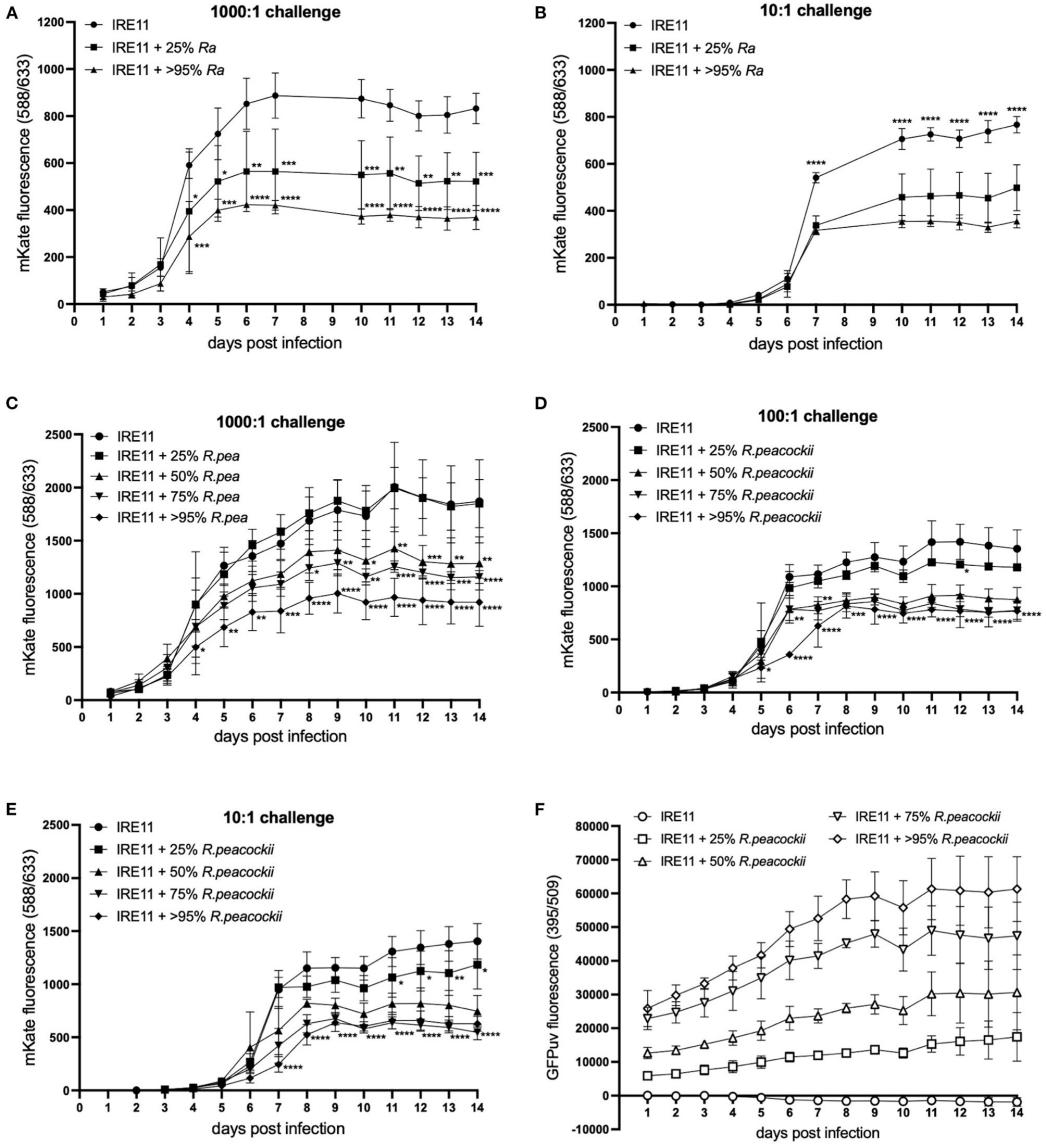
*Rickettsia parkeri* replication in the presence of *R. amblyommatis* or *R. peacockii*. Replication of *R. parkeri*-mKate in tick cells was monitored for 14 days by measuring mKate fluorescence on a microplate reader. **(A,B)** *R. parkeri-*mKate replication in IRE11 cells with or without *R. amblyommatis* at 28°C in a candle jar at challenge doses of 1,000:1 **(A)** and 10:1 **(B)**; readings not taken on day 8 or 9. **(C–E)** *R. parkeri-*mKate replication in IRE11 cells with or without *R. peacockii-*GFPuv at 28°C in a candle jar at challenge doses of 1,000:1 **(C)**, 100:1 **(D)**, and 10:1 **(E)**. **(F)** GFPuv fluorescence indicating replication of *R. peacockii-*GFPuv. Data show mean and error bars standard deviation of three replicate wells. Means were compared to the uninfected control IRE11 using a two-way ANOVA with Dunnett's multiple-comparison test; statistically significant values are marked by asterisks ^*^p < 0.05, ^**^p < 0.01, ^***^p < 0.001, ^****^p < 0.0001. Data are representative of two independent experiments (see Supplementary Figure S4).

In order to begin to separate whether the observed inhibition of pathogen growth might be due to antibiosis by *R. buchneri* or competitive exclusion as observed previously (70–73) between various species of *Rickettsia* (that do not contain antibiotic synthesis gene clusters), additional plate reader experiments were performed using different *Rickettsia* species in place of *R. buchneri* as the resident bacteria. Firstly, this was assessed using the low-pathogenic species *R. amblyommatis* at infection levels of 25 and >95% in IRE11 cells, and uninfected control IRE11, which were then challenged with *Rp-*mKate at 1,000:1 (high) and 10:1 (low). In contrast to results obtained with *Rb-*GFPuv, the presence of *R. amblyommatis* in tick cells resulted in only partial inhibition of *Rp-*mKate replication. Growth of *Rp-*mKate in *R. amblyommatis*-infected IRE11 was inhibited in a manner that was relative to the level of *R. amblyommatis* infection (Figures 6A,B); i.e., at the low level of infection (25%), there was a lower inhibition of *Rp-*mKate (35% inhibition at 10:1 challenge, and 37% at 1,000:1, compared to IRE11 without *R. amblyommatis* at day 14), while at the high level of infection (>95%) there was a higher inhibition of *Rp-*mKate (54% inhibition at 10:1 challenge and 56% at 1,000:1).

To investigate whether endosymbiotic rickettsiae may have greater exclusionary effect on pathogenic bacteria than other pathogenic *Rickettsia* species, as well as to further investigate the contribution of the involvement of potential antibiotic production by *R. buchneri*, additional plate reader competition assays were conducted using *R. peacockii* (an endosymbiont of *D. andersoni*) in place of *Rb-*GFPuv. Wells contained either uninfected IRE11 or IRE11 infected with *R. peacockii-*GFPuv at levels of 25, 50, 75, and >95%. The wells were challenged with *Rp-*mKate at 1,000:1, 100:1, and 10:1. In the high-challenge (1,000:1) wells, mKate fluorescence was significantly lower than that in IRE11 without *R. peacockii*-GFPuv at day 4 onward in cells infected with >95% *R. peacockii*-GFPuv (Figure 6C). mKate fluorescence in IRE11 with 75% or 50% *R. peacockii-*GFPuv was significantly lower from day 8 and day 10 onward, respectively, while growth of *Rp-*mKate in IRE11 with 25% *R. peacockii-*GFPuv was not significantly different from IRE11 without the endosymbiont (Figure 6C). At day 14, compared to that in IRE11 without *R. peacockii-*GFPuv, there was a 31% reduction in mKate fluorescence in IRE11 with 50% *R. peacockii-*GFPuv, a 38% reduction in IRE11 with 75% *R. peacockii-*GFPuv, and a 51% reduction in IRE11 with >95% *R. peacockii-*GFPuv. Similarly, in the 100:1 challenge wells, mKate fluorescence in 50, 75, and >95% *R. peacockii-*GFPuv-infected IRE11 was significantly different from that in IRE11 without *R. peacockii-*GFPuv, from day 5 (>95%) or day 6 (50 and 75%) onward, while mKate fluorescence in 25% *R. peacockii-*GFPuv-infected IRE11 was similar to the control (Figure 6D). At day 14, compared to that in IRE11 without *R. peacockii-*GFPuv, there were reductions of 35%, 43%, and 43% in mKate fluorescence measured in IRE11 with 50, 75, and >95% *R. peacockii-*GFPuv, respectively. In the 10:1 challenge experiment, mKate fluorescence was significantly reduced in IRE11 50, 75, and >95% infected with *R. peacockii-*GFPuv relative to IRE11 without *R. peacockii-*GFPuv from day 7 onward (Figure 6E). In IRE11 25% infected with *R. peacockii-*GFPuv, mKate fluorescence was significantly lower than the control from days 11 to 14 (Figure 6E). At day 14, in comparison to that in IRE11 without *R. peacockii-*GFPuv, mKate fluorescence was reduced by 16, 47, 61, and 55% in IRE11 with 25, 50, 75, and >95% *R. peacockii-*GFPuv, respectively. Measurement of GFPuv fluorescence could differentiate the different populations of IRE11 infected with 25, 50, 75, and >95% *R. peacockii-*GFPuv and implied a steady replication of *R. peacockii-*GFPuv over time (Figure 6F). No increase in GFPuv fluorescence was observed in uninfected IRE11; rather, there was a decline, likely due to lysis of IRE11 by *Rp-*mKate in infected cells (Figure 6F). Together, these results suggest that neither *R. amblyommatis* nor *R. peacockii* possess the ability to inhibit growth of competing *R. parkeri* to the same extent as *R. buchneri*.

**Figure 7:**
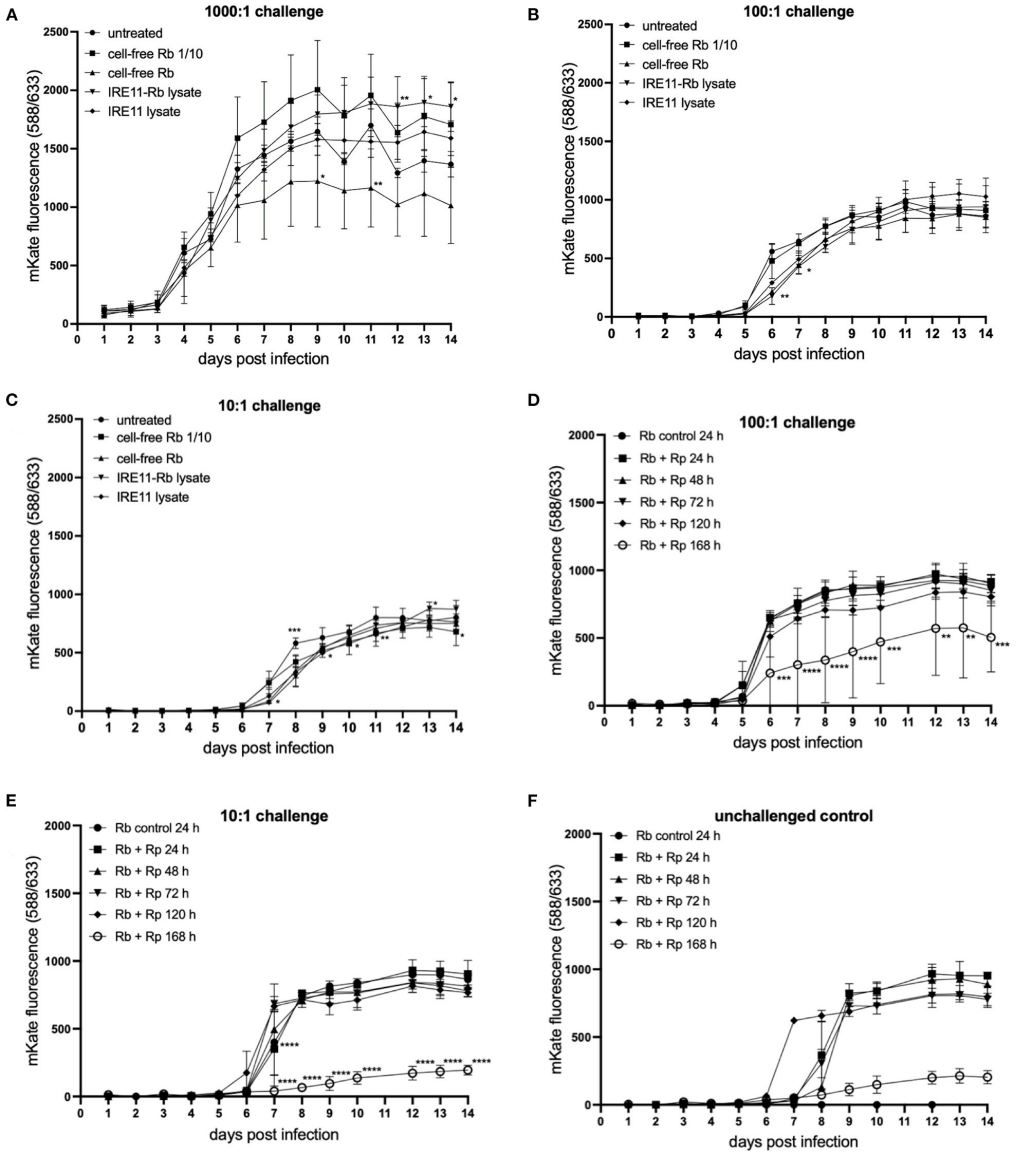
*Rickettsia parkeri* replication in IRE11 treated with cell-free *R. buchneri* or *R. buchneri* lysate. Replication of *R. parkeri*-mKate in tick cells was monitored for 14 days by measuring mKate fluorescence on a microplate reader. **(A–C)** *R. parkeri-*mKate replication in IRE11 at challenge doses of 1,000:1 **(A)**, 100:1 **(B)**, and 10:1 **(C)** in the presence of cell-free *R. buchneri*, lysate from *R. buchneri-*infected IRE11, or lysate from uninfected IRE11. **(D–F)** *R. parkeri-*mKate replication in IRE11 treated with lysates from *R. buchneri* challenged with *Rp-*mKate for 24, 48, 72, 120, or 168 h. Treated cells were challenged with 100:1 **(D)** or 10:1 **(E)** *Rp-*mKate. Unchallenged control indicates that viable *Rp-*mKate are present in cell lysate **(F)**. Data show mean and error bars standard deviation of three replicate wells. Means were compared to the uninfected control IRE11 using a two-way ANOVA with Dunnett's multiple-comparison test; statistically significant values are marked by asterisks ^*^p < 0.05, ^**^p < 0.01, ^***^p < 0.001, ^****^p < 0.0001.

Further experiments were performed to examine whether the inhibitory effect of *R. buchneri* on *Rp-*mKate infection was due to the secretion of antibiotic products. Uninfected IRE11 was added to a 96-well plate then treated with either cell-free *Rb*-WT, a 1:10 dilution of cell-free *Rb-*WT, lysate from IRE11 heavily infected with *Rb-*WT, or lysate from uninfected IRE11. After 2 h, cells were challenged with *Rp-*mKate. Although there were some significant differences in the growth of *Rp-*mKate under some of the treatments, these were inconsistent across the three different challenge doses, suggesting that rather they were due to experimental variations, for example in challenge dose or cell density (Figures 7A–C). Taking these data together, there seems to be no obvious effect of any of the treatments on the growth of *R. parkeri* in comparison to untreated control cells. Furthermore, these results suggest that *R. buchneri* need to be intracellular to inhibit *R. parkeri* growth and that there is also no antibiotic effect of extracellular addition of *Rb-*WT lysate.

An additional experiment was performed to determine whether challenging *R. buchneri-*infected IRE11 with *Rp-*mKate would increase the inhibitory activity of lysates against *Rp-*mKate. Lysates were prepared from cell-free *Rb-*WT isolated from IRE11 challenged with *Rp-*mKate for 1–7 days and then used to treat IRE11 cells in a 96-well plate. The wells were then challenged as previously with *Rp-*mKate at challenge doses of 1,000:1, 100:1, and 10:1, and mKate fluorescence was measured over 14 days and compared to mKate fluorescence in cells treated with lysate from unchallenged *Rb-*WT-infected IRE11. At 1,000:1 challenge, there was no difference in mKate fluorescence between the control and any of the treatments (Supplementary Figure S5), showing a growth curve similar to that in other plate reader experiments. However, at challenge doses of 100:1 and 10:1, there were significant differences in mKate fluorescence in the wells treated with lysates from *Rb-*WT challenged with *Rp-*mKate for 7 days (168 h) in comparison to the control, with 43 and 77% reductions at day 14, respectively (Figures 7D,E). The mKate fluorescence in the other treatment groups was similar to that in the control wells. Unchallenged control wells were also incorporated into the experiment to check the viability of any rickettsiae in the lysate; mKate fluorescence was observed increasing on days 6–8, suggesting that viable *Rp-*mKate were present in the lysate (Figure 7F). However, their growth in wells treated with 168-h-challenged lysate was also much lower than in other treatment wells. The fact that the mKate fluorescence in the unchallenged control wells peaked later than in challenged wells suggests that the low level of viable *Rp-*mKate contributed little to the results seen in the *Rp-*mKate-challenged wells. Overall, these results indicate that after 7 days in the presence of *Rp-*mKate, lysates from *Rb-*WT were able to inhibit the replication of *Rp-*mKate in IRE11 cells at lower doses (100:1 and 10:1), which could be due to increased antibiotic activity at this time point.

### Expression of Antibiotic Genes in Response to *R. parkeri* Infection

**Figure 8:**
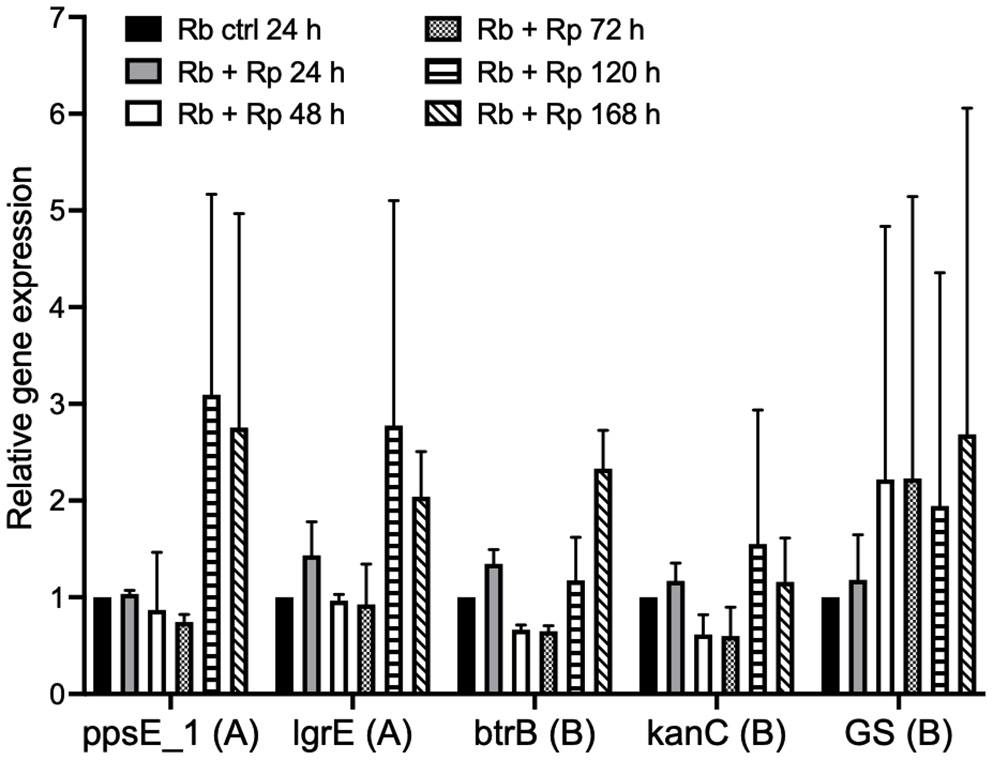
Expression of *R. buchneri* antibiotic cluster genes in response to *Rickettsia parkeri* challenge. Relative expression of *R. buchneri* antibiotic cluster genes in response to *R. parkeri-*mKate challenge over 7 days. Bars show the expression of each gene relative to the control (unchallenged *R. buchneri*) as the mean and standard deviation of two independent experiments for each qRT-PCR assay. Ct values were normalized to *GAPDH* expression in infected IRE11 cells. Genes from the polyketide and aminoglycoside clusters are denoted by (A) and (B), respectively. Means were compared to the unchallenged control using a two-way ANOVA with Dunnett's multiple-comparison test.

To investigate whether the expression of genes from the putative antibiotic clusters of *R. buchneri* was upregulated in response to the presence of potentially competing bacteria, a time-course experiment was set up and qRT-PCR was used to examine the relative expression of selected genes from each antibiotic cluster in the presence and absence of *Rp-*mKate during infection. Over a 7-day time course, several of the examined genes appeared to be upregulated in response to multiple days' challenge with *Rp-*mKate (Figure 8); however, there was wide variation between replicate experiments and the differences were not statistically significant.

### Investigation of *R. buchneri* Antibiotic Activity

The antibiotic activity of *R. buchneri* against extracellular bacteria was investigated using antibiotic susceptibility assays. Filter paper disks were treated with cell-free *Rb-*WT, *Rb-*WT-infected IRE11, and supernatant from IRE11 cultures infected with various levels of *Rb-*WT (25%, 50%, and >95%). Spectinomycin (10 and 100 μg)-treated disks were used as positive controls, and uninfected IRE11 cells and IRE11 culture supernatant were used as negative controls. Disks were placed onto Mueller–Hinton agar plates streaked with *E. coli* strain D21 or *S. aureus* strain MN8. None of the IRE11- or *Rb-*WT-derived treatments resulted in any inhibition of *E. coli* or *S. aureus* growth, whereas spectinomycin-treated disks prevented bacterial growth (Table 4).

**Table 4:** Results from antibiotic susceptibility tests (disk diffusion assays) against *E. coli* and *S. aureus*.

	***E. coli* D21**	***S. aureus* MN8**
Experiment 1—cell lysates		
Cell-free Rb	-	-
Rb-infected IRE11	-	-
Uninfected IRE11	-	-
Spectinomycin 10 μg	+	+
Spectinomycin 100 μg	+	+
Experiment 2—live cells and supernatant		
Cell-free Rb	-	-
IRE11 + 25% Rb	-	-
IRE11 + 50% Rb	-	-
IRE11 + >95% Rb	-	-
Uninfected IRE11	-	-
IRE11 + 25% Rb supernatant	-	-
IRE11 + 50% Rb supernatant	-	-
IRE11 + >95% Rb supernatant	-	-
IRE11 supernatant	-	-
Spectinomycin 100 μg	+	+

*+ inhibition of bacterial growth; - no inhibition of bacterial growth*.

Supernatant from IRE11 heavily infected with *Rb-*WT was also tested for activity against *Rp-*mKate growing in Vero cells. Addition of the supernatant to Vero cultures prior to infection with *Rp-*mKate did not inhibit infection; there were no differences observed in the progression of plaque formation and size of plaques in comparison to a duplicate experiment using supernatant from an uninfected IRE11 culture (Supplementary Figure S6).

## Discussion

There is growing evidence that competition between endosymbiotic and pathogenic *Rickettsia* species in tick vectors may play an important role in the persistence and transmission of rickettsial pathogens (70–73). This study describes the existence of two putative antibiotic synthesis gene clusters in the genome of the *I. scapularis* endosymbiont *R. buchneri* isolated in Minnesota. Furthermore, *in vitro* experiments show that *R. buchneri* exerts an inhibitory effect on the growth of pathogenic rickettsiae in tick cell culture. Inhibition of *R. parkeri* growth by *R. buchneri* was greater than that exhibited by the other rickettsiae examined in this study, the low pathogenic *R. amblyommatis*, and the endosymbiont *R. peacockii*, which may suggest that this is due to the presence of antibiotic synthesis genes in *R. buchneri* that are lacking in other rickettsiae. Even at low infection rates, the presence of *R. buchneri* resulted in significant perturbation of *R. parkeri* growth, while the presence of either *R. amblyommatis* or *R. peacockii* showed a more direct competition where higher infection rates lead to a greater reduction in the growth of *R. parkeri*. These results correlate with what is known from current field and laboratory data, which suggest reduced horizontal and vertical transmission of *R. parkeri* or *R. rickettsii* by ticks in the presence of coinfecting *R. amblyommatis* or *R. peacockii* (70, 72, 73) and an almost complete absence of any coinfecting *Rickettsia* species in *R. buchneri-*infected *I. scapularis*. While a link between this inhibition and antibiotic synthesis is yet to be proven, this work raises the possibility that antibiotic production by *R. buchneri* may be a mechanism for exclusion of competing intracellular bacteria from its host tick.

Although genes from the clusters are actively transcribed by *R. buchneri*, no evidence of antibiotic activity was found in lysates or supernatants from *R. buchneri-*infected cultures, against either *R. parkeri* or the extracellular bacteria *E. coli* and *S. aureus*. However, when grown in the presence of *R. parkeri* for 7 days, lysates from *R. buchneri-*infected cells showed some inhibitory activity against lower challenge doses (100:1 and 10:1) of *Rp-*mKate, suggesting that *R. parkeri* challenge could be a trigger that might induce antibiotic activity of *R. buchneri*. However, results from qRT-PCR examining the expression of certain genes in the clusters in the presence of *R. parkeri* were highly variable and therefore inconclusive. Further analyses to obtain additional information about the regulation of the antibiotic synthesis clusters are required to understand how they respond to *R. parkeri* challenge. For example, it is unknown whether the clusters are transcribed as operons or in what ratios the different components of the clusters are required for antibiotic production. The antibiotic compounds produced by *R. buchneri* might only act intracellularly to prevent cells inhabited by the endosymbiont being invaded by other bacteria which could compete for resources. However, the fact that even when only a quarter of cells are occupied by *R. buchneri* results in inhibition of *R. parkeri* growth suggests that there must be some effect of *R. buchneri* on neighboring cells, which could potentially be mediated through the delivery of compounds to adjacent cells that are either antimicrobial or make cells refractory to infection. Further in-depth studies are required to elucidate the mechanisms of anti-rickettsial activity as well as how the putative compounds may be transported.

The proteins encoded by the aminoglycoside gene cluster show similarity to those from Actinobacteria, particularly *Streptomyces* spp. and Firmicutes (54), while the polyketide gene cluster encodes proteins with similarity to those of Gammaproteobacteria, particularly *Erwinia amylovora, Pantoea ananatis*, and *Legionella* spp. This suggests these gene clusters were likely obtained from environmental bacteria; indeed, members of these phyla have been identified in the *I. scapularis* microbiome (21, 22, 24, 32, 92). The *R. buchneri* genome is known to be highly plastic and contains multiple mobile genetic elements and sections of genetic material from other microorganisms (54). As ticks spend a large portion of their life in the environment in close association with soil, leaf litter, and vegetation, and also come into contact with the skin and blood of animals during host-seeking and feeding, it is likely that they encounter numerous environmental bacteria from which these gene clusters could have been transferred. Further examination of the gene clusters and similar pathways in the bacterial taxa from which these most likely originated may give us a better understanding of the likely antimicrobial compounds that could be synthesized by *R. buchneri*, and this could lead to future isolation and characterization of these products and determination of their antimicrobial activity. For example, *Legionella* spp. are known to contain multiple gene clusters for synthesis of polyketides/non-ribosomal peptides (93), and polyketide synthases were identified in *Pantoea* and *Erwinia* species that showed antagonistic activity (attributed to antibiosis) against the rice pathogen *Xanthomonas oryzae* (94). Similarly, strains of *P. ananatis* and *P. agglomerans* contain antibiotic biosynthesis clusters that allow them to compete with *E. amylovora* (95, 96). Interestingly, the *Rb* ISO7 polyketide cluster includes a putative type IV pilin; these proteins have diverse functions including adherence, motility, biofilm formation, host cell manipulation, DNA transfer, and protein secretion (97). It will be interesting to further investigate the role of this protein in *R. buchneri*, as many functions of this class of pilins could be related to the antibiotic activity of the cluster. Most genes in the polyketide cluster are absent from the REIS (Wikel) genome, and there may have been some recombination of this region resulting in excision of a large portion of the cluster that is found in *Rb* ISO7. This might suggest that this gene cluster is not essential for the endosymbiont, and the lack of antimicrobial products it synthesizes is compensated for by the presence of the aminoglycoside cluster. The REIS (Wikel) genome is derived from that of a lab colony of *I. scapularis*, whereas the *Rb* ISO7 genome originates from a field-collected tick, so an alternative explanation for the loss of these genes in REIS (Wikel) is that they are not essential for survival in the lab but could be necessary under natural conditions for protection against challenge from environmental microbes, for example. Further experiments to compare *R. buchneri* with REIS (Wikel) might determine whether absence of the polyketide cluster has any effects on survival or competition with other bacteria.

Given the inhibitory effect of *R. buchneri* on the *in vitro* infection and replication of the intracellular pathogens *A. phagocytophilum, R. monacensis*, and *R. parkeri*, one role of the endosymbiont may be the exclusion of pathogens from the tick. As *R. buchneri* is primarily restricted to the ovaries of female ticks, in nature it might be involved in preventing the colonization of this organ and subsequent transovarial transmission of intracellular pathogens, such as other *Rickettsia* species. While inhibition of *A. phagocytophilum* was seen in tick cell culture, it is unclear whether *R. buchneri* has any effect on *A. phagocytophilum* infection within the tick vector. Sakamoto *et al*. found that *A. phagocytophilum* levels were higher in male ticks, which have significantly lower titers of *R. buchneri* (19), while Steiner *et al*. found no correlation between *R. buchneri* and *A. phagocytophilum* infection prevalence (48), yet few other studies have examined interactions between these two bacteria in tick populations. Similarly, there is little data on whether *R. buchneri* has any effect on *I. scapularis* infection with the spirochete *B. burgdorferi*. Steiner and colleagues found that *B. burgdorferi* infection rates were significantly higher when *R. buchneri* was not detected, but only for male *I. scapularis* (48). One microbiome study found that *Rickettsia* reads were significantly less abundant in *B. burgdorferi*-positive ticks (98), while another found no differences in bacterial composition between *B. burgdorferi-positive* and negative ticks, for either males or females (20). Neither of these pathogens are transovarially transmitted, with *A. phagocytophilum* localizing to the salivary glands (99) and *B. burgdorferi* residing in the midgut (100). That both pathogens are highly prevalent in *I. scapularis* populations supports the hypothesis that any antibiotic activity *R. buchneri* may have is likely restricted to the ovaries. In this study, *R. buchneri* inhibition of tick-borne pathogens was observed in a tick cell culture system that is likely not directly comparable to life inside the tick. Further studies examining the effect of *R. buchneri* on infection by these pathogens using *ex vivo* ovaries (101) and/or live *I. scapularis* should provide additional insights into the dynamics of this competition. With the ability to remove *R. buchneri* from ticks with the use of ciprofloxacin (26), *in vivo* studies to further examine the consequences for ticks lacking the endosymbiont and whether they are susceptible to infection with pathogenic rickettsiae can be performed in future.

*Ixodes pacificus*, the main tick vector of *B. burgdorferi* and *A. phagocytophilum* in the western US, also harbors a highly prevalent rickettsial endosymbiont, “*R. monacensis*” strain Humboldt (56, 102, 103), and is not known to vector pathogenic rickettsiae. However, application of genome similarity sequence-threshold criteria indicates that this endosymbiont is a new distinct species closely related to *R. buchneri* and *R. monacensis* (104). In addition, *I. pacificus* is often coinfected with *Rickettsia* phylotype G022 (102, 103). While little is currently known about phylotype G022, it is more closely related to pathogenic SFG rickettsiae than to *R. buchneri* and *Rickettsia* strain Humboldt (103). Cheng et al. suggested that it is likely to be the “Tillamook agent” previously isolated from *I. pacificus* and shown to be mildly pathogenic in guinea pigs (105, 106). However, this agent has recently been characterized and found to be a separate species (*R. tillamookensis* sp. nov.) related to the transitional group of *Rickettsia* (107), meaning that *I. pacificus* is associated with two potentially pathogenic rickettsiae in addition to its endosymbiont. The *Rickettsia* strain Humboldt genome (NZ_LAOP01000001.1) does not appear to contain antibiotic gene clusters similar to those found in *R. buchneri*, which might be one reason that *I. pacificus* can be coinfected with both its endosymbiont and potentially pathogenic species. Interestingly, field-collected *I. scapularis* have occasionally been found containing *R. amblyommatis, R. montanensis*, or *R. parkeri* (25, 31, 36, 41, 42, 44, 53, 108), which could potentially occur through “spillover” from host feeding alongside infected *A. americanum* or *D. variabilis*; however, these infected *I. scapularis* appear to be individuals lacking *R. buchneri* since the endosymbiont was not detected in these ticks. Only one coinfection of *I. scapularis* with *R. buchneri* and *R. parkeri* has been reported (44), and this was in a blood-fed tick collected from Louisiana black bears (*Ursus americanus luteolus*) also being fed on by *R. parkeri-*infected *A. maculatum*, making it likely that the pathogen was present in the infected blood meal.

Interference between rickettsiae has been little studied since it was proposed 40 years ago, but existing research shows that infection with a first *Rickettsia* species may reduce transovarial transmission of a second *Rickettsia* (70, 71, 73), reduce acquisition of the second *Rickettsia* from infected hosts, and/or reduce its replication in the tick (72, 73), all of which could potentially lead to reduced transmission of pathogenic rickettsiae in enzootic cycles. In this study, the presence of either *R. amblyommatis* or *R. peacockii* led to a reduction in the ability of *R. parkeri* to infect and replicate in tick cells, reflecting what has been observed in *in vivo* studies in ticks (70–73). However, the mechanisms by which this interference occurs remain unexplored. One potential mechanism that has been suggested is immune priming (26), in which extracellular *Rickettsia* could stimulate the tick innate immune response, making the vector less susceptible to infection with a second *Rickettsia*. Symbionts have been shown to be important for immune development and protection from pathogens in other arthropods (109–111). It is also possible that occupation of tick cells or tissues by one *Rickettsia* species could physically prevent them being infected by a second *Rickettsia*, reducing their ability to effectively spread and replicate in the tick.

In summary, this research provides evidence that the endosymbiont of *I. scapularis, R. buchneri*, exerts an inhibitory effect on the growth of pathogenic tick-borne bacteria in cell culture and possesses two gene clusters encoding putative antibiotic biosynthesis machinery. This might suggest that besides being a potential nutritional endosymbiont, *R. buchneri* could also provide the service of preventing pathogenic *Rickettsia* species from occupying the ovaries, which could be detrimental to the tick's biology as has been shown to be the case for *R. rickettsii* in *D. andersoni* and *D. variabilis* (112, 113). While a correlation between the presence of antibiotic clusters and the ability to inhibit the growth of pathogenic Rickettsiae was found in this study, confirmation that the observed inhibition is directly linked to *R. buchneri*'s antibiotic clusters requires further investigation. Supportive evidence from *in vivo* studies could have important implications for our understanding of rickettsial interference and the vector competence of *I. scapularis* for SFG rickettsiae.

## Data Availability Statement

The datasets generated for this study can be found in the Data Repository for the University of Minnesota, https://doi.org/10.13020/ZQXG-JF78.

## Author Contributions

BC, JO, TK, and UM conceived and designed the experiments. BC, NB, TK, and X-RW performed the experiments. BC and TK analyzed the data. CT developed the fluorescent plate reader assay. BC drafted the manuscript and prepared the figures. All authors contributed to the manuscript revision and approved the submitted version.

## Funding

This research was supported by generous funding from the National Institutes of Health grants R01 AI49424 and R01 AI081690 to UM (http://www.grants.nih.gov/grants/oer.htm) and funds from the University of Minnesota Agricultural Experiment Station. The funders had no role in the study design, data collection, and analysis, decision to publish, or preparation of the manuscript.

## Conflict of Interest

The authors declare that the research was conducted in the absence of any commercial or financial relationships that could be construed as a potential conflict of interest.

## Publisher's Note

All claims expressed in this article are solely those of the authors and do not necessarily represent those of their affiliated organizations, or those of the publisher, the editors and the reviewers. Any product that may be evaluated in this article, or claim that may be made by its manufacturer, is not guaranteed or endorsed by the publisher.
