# Supplementary material for: The Ixodes scapularis Symbiont Rickettsia buchneri Inhibits Growth of Pathogenic Rickettsiaceae in Tick Cells: Implications for Vector Competence
Source: Front Vet Sci. 2022 Jan 6;8:748427. doi: 10.3389/fvets.2021.748427 (PMC8770908; doi:10.3389/fvets.2021.748427)
Supplement: Supplementary file 1 [file Data_Sheet_1.PDF]

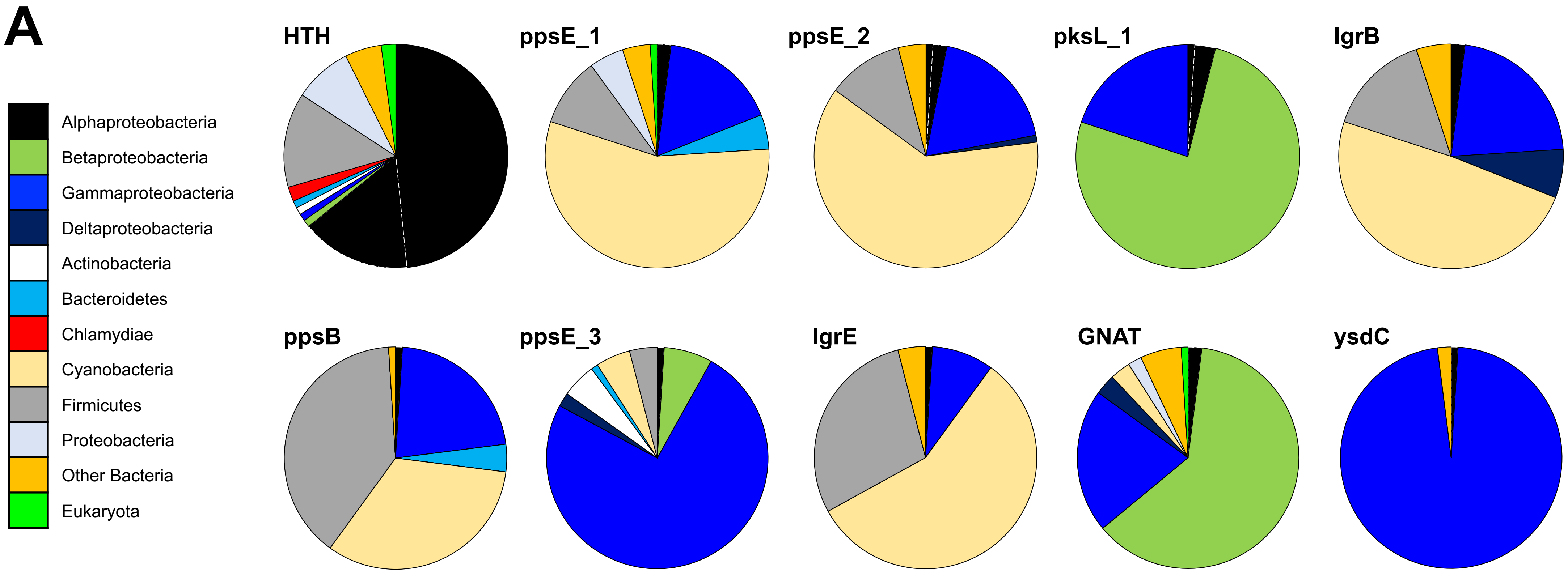

B

Rb ISO7	HTH	pilin	ppsE_1	ppsE_2	pksL_1	lgrB	ppsB	ppsE_3	lgrE	GNAT	ysdC
**REIS (Wikel)**	100	100	99.7*	*absent*	*absent*	*absent*	*absent*	*absent*	*absent*	*absent*	*absent*

C

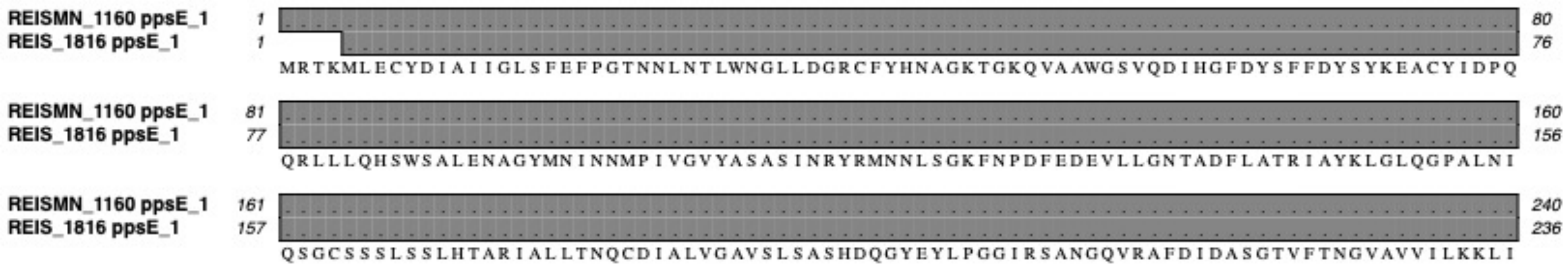

Figure S1. (A) Pie charts showing taxa of top 100 blastp hits from each protein in the polyketide synthesis cluster using Rb ISO7 sequences as queries. The Rickettsiales are separated from other Alphaproteobacteria by dashed white lines. No graph is shown for type IV pilin because the only 2 hits were from *R. buchneri* genomes. Full results can be found in Supplementary Data 1.

(B) Table showing the identity (%) of polyketide cluster amino acid sequences from the REIS (Wikel) genome to those from the *R. buchneri* ISO7 genome. * Predicted start codon is later in REIS ppsE_1, otherwise amino acid sequence is identical to that of *Rb* ISO7; an alignment of the first 240 amino acids of the proteins is shown in (C).

A

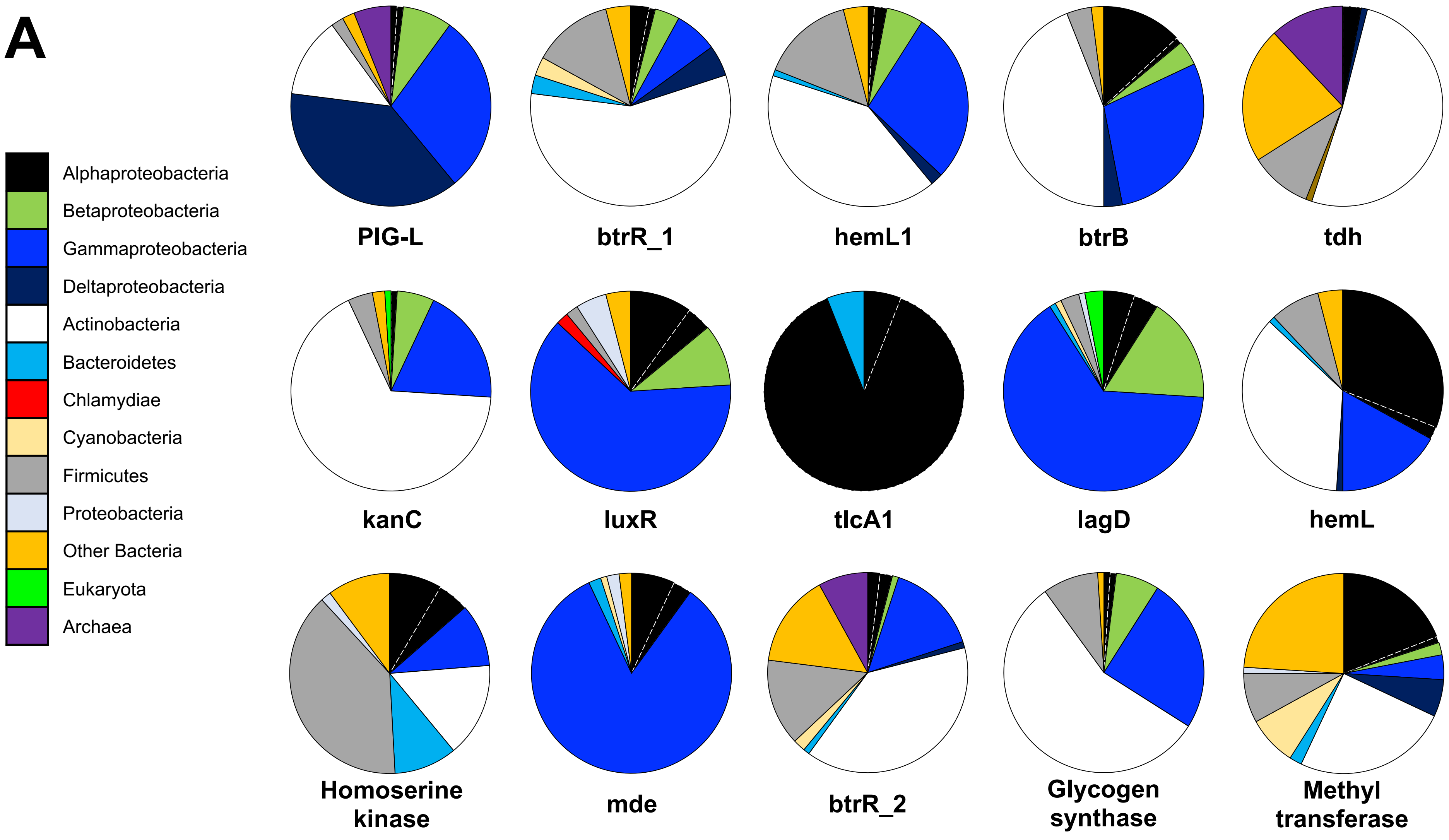

B

REIS (Wikel)	PIG-L	KanB	HemL1	Sis6	tdh	IstC	LuxR	Tlc2	ABC	HemL	AprU	mdlB	btrR	IstM	IstN
Rb ISO7	100	100	100	100	84.8*	100	100	100	100	99.5	95.0*	99.7	100	100	100

C

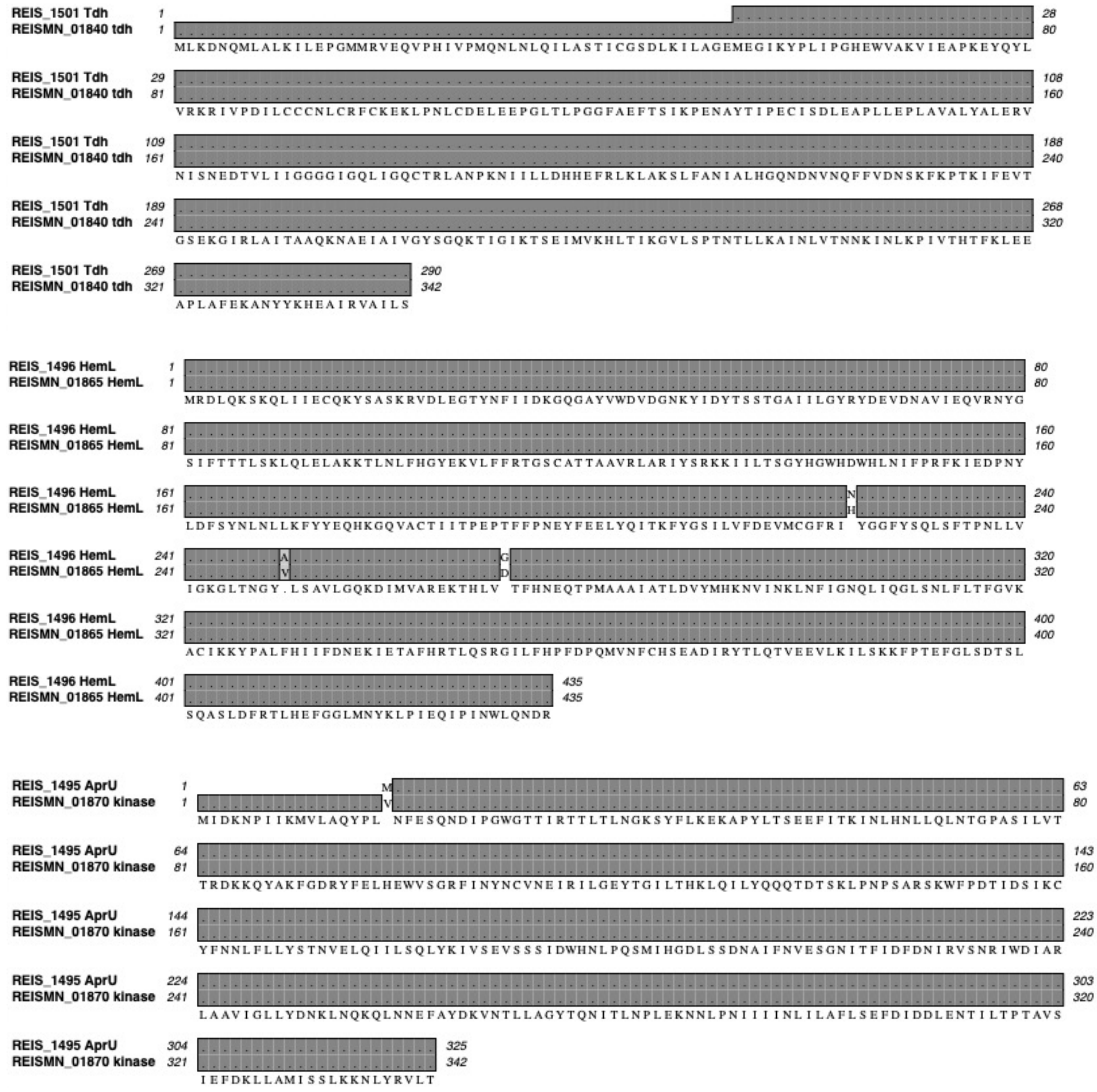

Figure S2. (A) Pie charts showing taxa of top 100 blastp hits from each protein in the aminoglycoside synthesis cluster using Rb ISO7 sequences as queries. The Rickettsiales are separated from other Alphaproteobacteria by dashed white lines. Full results can be found in Supplementary Data 2.
(B) Table showing the identity (%) of aminoglycoside cluster amino acid sequences from the *R. buchneri* ISO7 genome to those from the REIS (Wikel) genome. * Predicted start codon is later in REIS (Wikel) tdh and AprU, otherwise amino acid sequence is identical to those of *Rb* ISO7. (C) Alignments of amino acid sequences of tdh, HemL, and AprU.

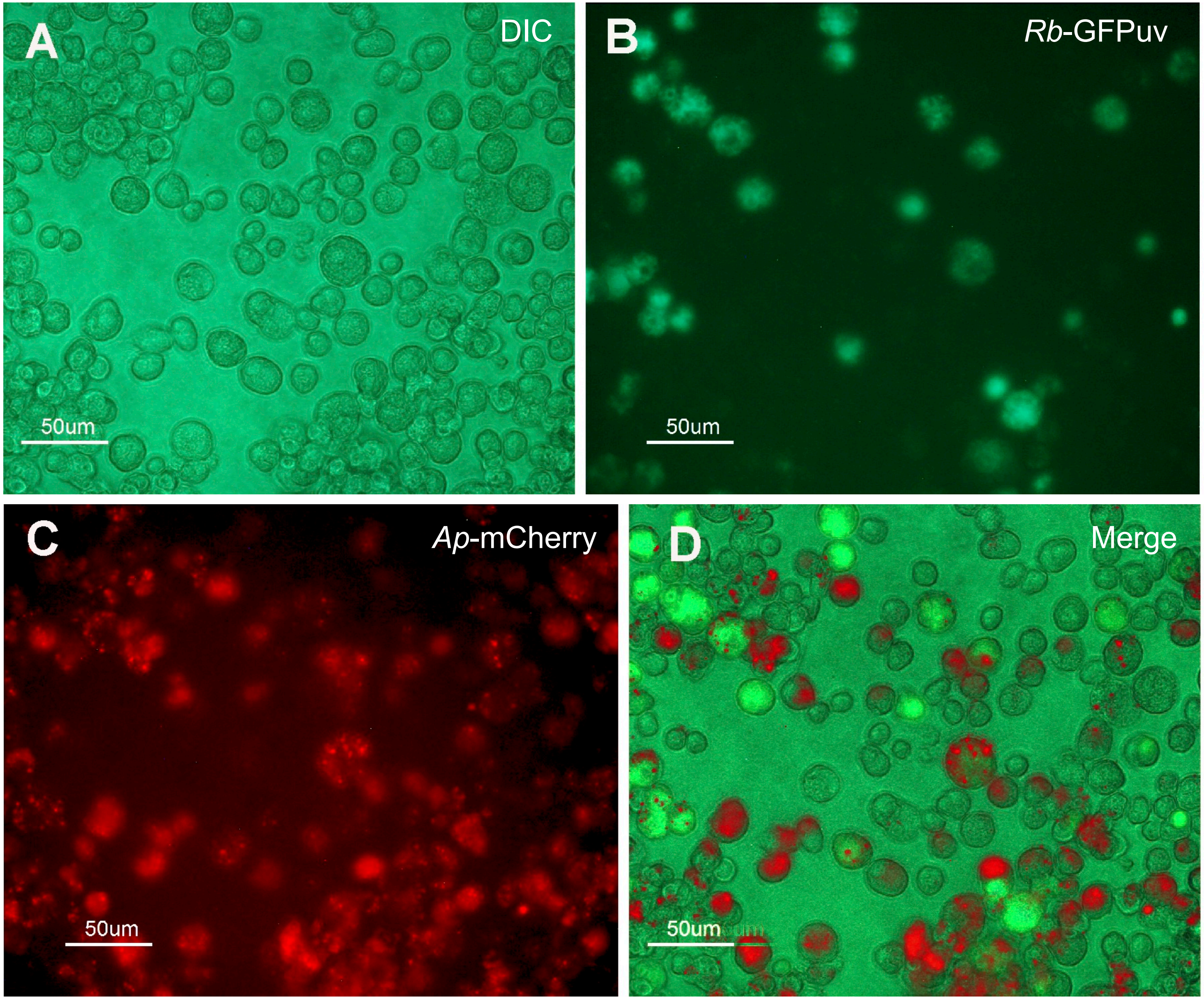

Figure S3. Images of IRE11 cell layer with ~25% of the cells infected with *R. buchneri*-GFPuv and challenged with *A. phagocytophilum*-mCherry*.* Live cell images were captured on a Nikon Diaphot fluorescent microscope using FITC and TRITC filters.

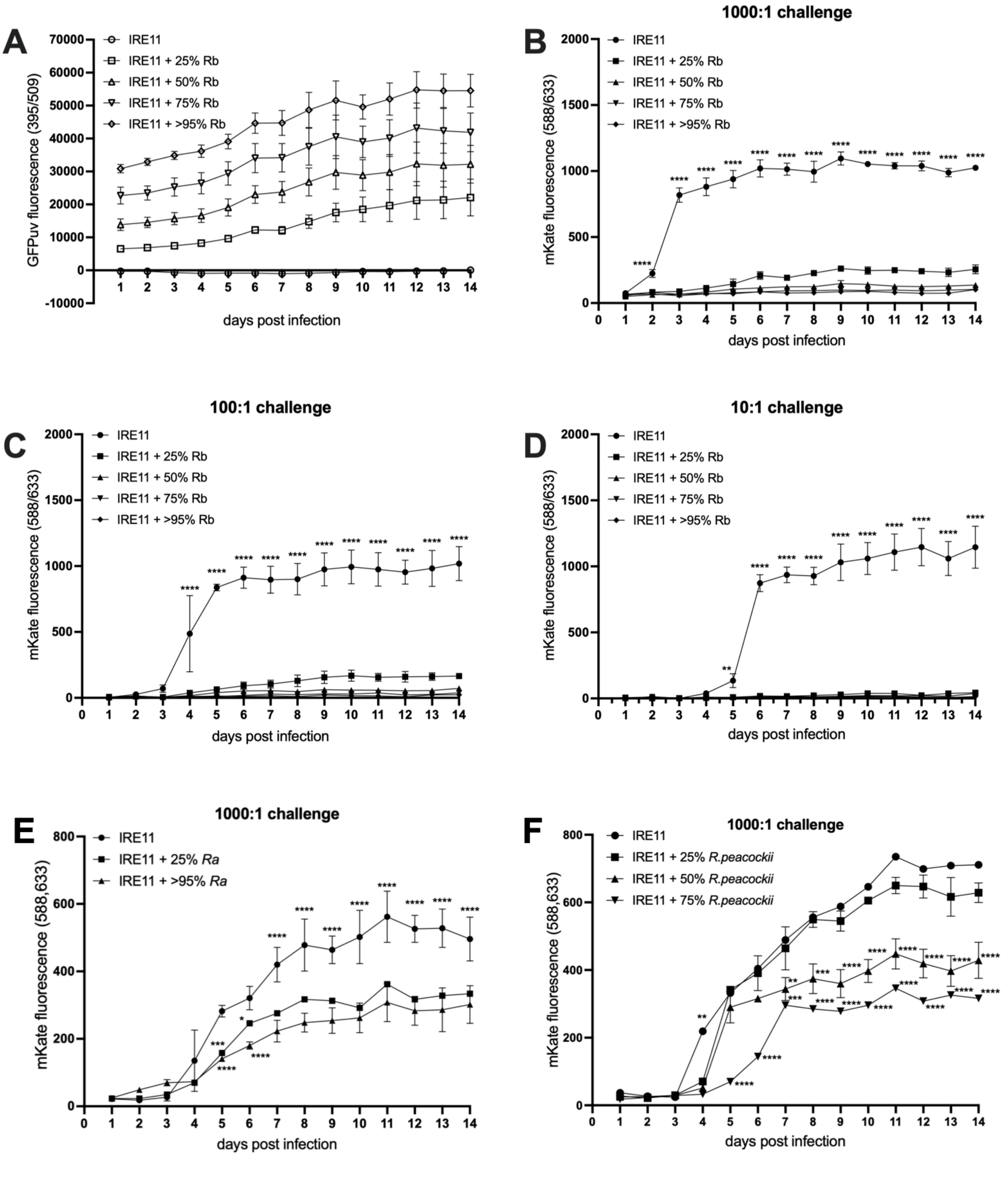

Figure S4. *Rickettsia parkeri* replication in IRE11 in the presence of *R. buchneri* (A – D)*, R. amblyommatis* (E) or *R. peacockii* (F) - repeat fluorescent plate reader experiments. Rickettsial replication in IRE11 cells was monitored for 14 days by measuring GFPuv and/or mKate fluorescence on a microplate reader. (**A**) GFPuv fluorescence indicating replication of *R. buchneri*-GFPuv. (**B-D**) mKate fluorescence indicating growth of *R. parkeri*-mKate at challenge doses of 1000:1 (**B**), 100:1 (**C**), and 10:1 (**D**). mKate fluorescence in IRE11 cells infected with *R. amblyommatis* (**E)** or *R. peacockii*-GFPuv (**F**) challenged with 1000:1 *R. parkeri*-mKate. Lines show mean and error bars standard deviation of three replicate wells. Means were compared to the uninfected control IRE11 using a two-way ANOVA with Dunnett's multiple comparison test; statistically significant values are marked by asterisks * $p<0.05$, ** $p<0.01$, *** $p<0.001$, **** $p<0.0001$.

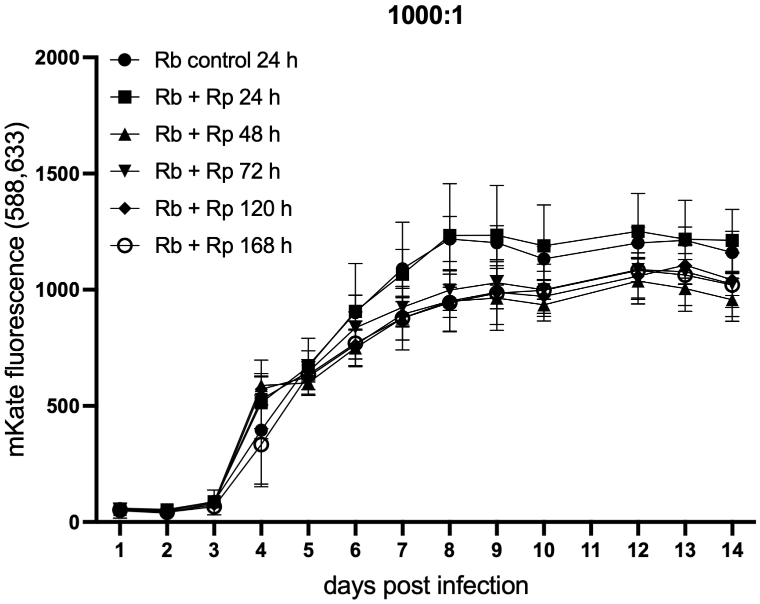

Figure S5. *R. parkeri*-mKate replication in IRE11 treated with lysates from *R. buchneri* challenged with *Rp*-mKate for 24, 48, 72, 120 or 168 hours. Treated cells were challenged with 1000:1 *Rp*-mKate. Data show mean and error bars standard deviation of three replicate wells.

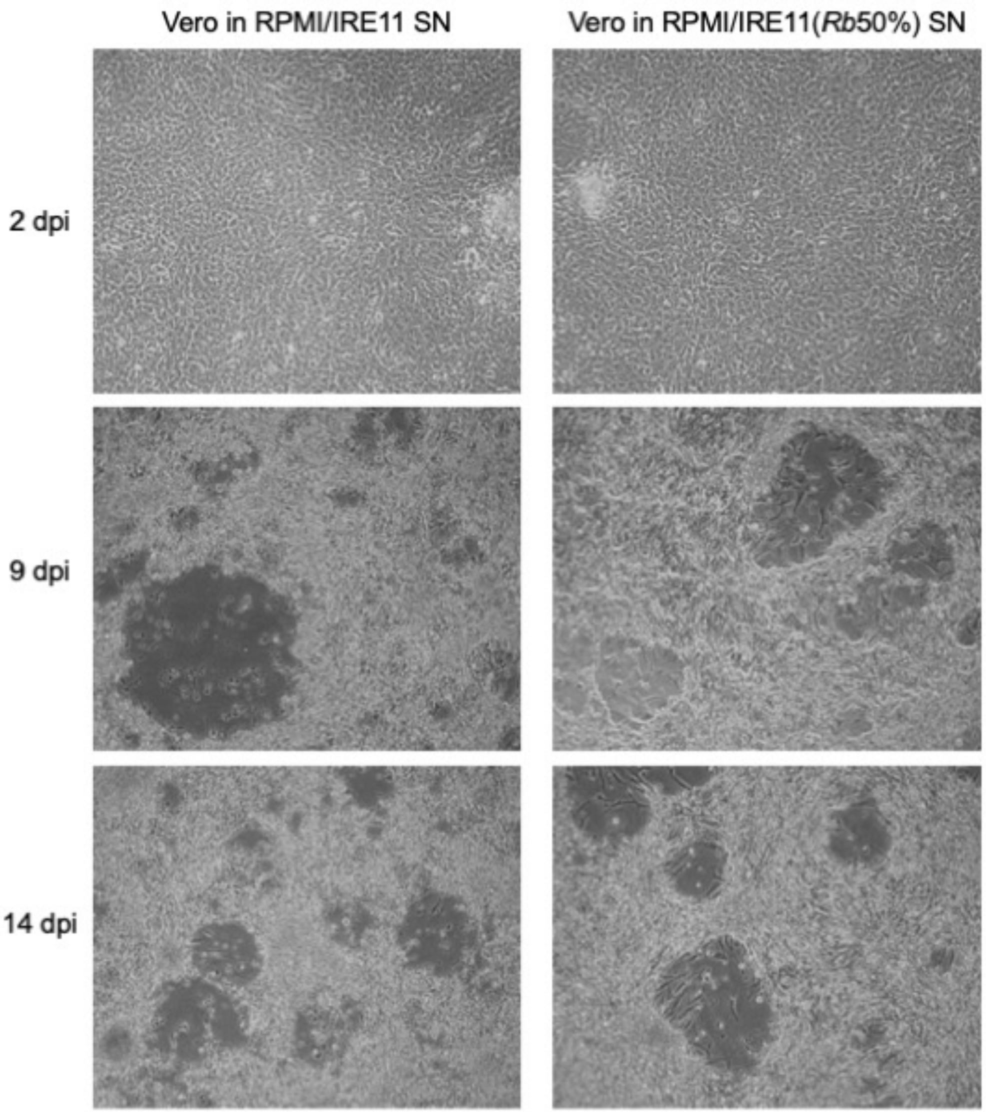

Figure S6. Cell culture supernatant from *Rickettsia buchneri*-infected IRE11 cells has no inhibitory effect on the infection of Vero cells by *R. parkeri*-mKate. Live cell images were captured on a Nikon Diaphot fluorescent microscope using brightfield filter.
